# High Quantum Yields and Biomedical Fluorescent Imaging Applications of Photosensitized Trivalent Lanthanide Ion-Based Nanoparticles

**DOI:** 10.3390/ijms252111419

**Published:** 2024-10-24

**Authors:** Tirusew Tegafaw, Dejun Zhao, Ying Liu, Huan Yue, Abdullah Khamis Ali Al Saidi, Ahrum Baek, Jihyun Kim, Yongmin Chang, Gang Ho Lee

**Affiliations:** 1 Department of Chemistry, College of Natural Sciences, Kyungpook National University, Taegu 41566, Republic of Korea; tirukorea@gmail.com (T.T.); djzhao.chem@gmail.com (D.Z.); ly1124161@gmail.com (Y.L.); yuehuan888@gmail.com (H.Y.); abdullah_al_saidi@hotmail.com (A.K.A.A.S.); 2 Department of Medical & Biological Engineering, Kyungpook National University, Taegu 41944, Republic of Korea; baxun@naver.com; 3 Department of Chemistry Education, Teachers’ College, Kyungpook National University, Taegu 41566, Republic of Korea; jkim23@knu.ac.kr; 4 Department of Molecular Medicine, School of Medicine, Kyungpook National University, Taegu 41944, Republic of Korea

**Keywords:** Ln^3+^ ion-based nanoparticle, photosensitization, host matrix, organic ligand, high quantum yield, biomedical fluorescent imaging application

## Abstract

In recent years, significant advances in enhancing the quantum yield (QY) of trivalent lanthanide (Ln^3+^) ion-based nanoparticles have been achieved through photosensitization, using host matrices or capping organic ligands as photosensitizers to absorb incoming photons and transfer energy to the Ln^3+^ ions. The Ln^3+^ ion-based nanoparticles possess several excellent fluorescent properties, such as nearly constant transition energies, atomic-like sharp transitions, long emission lifetimes, large Stokes shifts, high photostability, and resistance to photobleaching; these properties make them more promising candidates as next-generation fluorescence probes in the visible region, compared with other traditional materials such as organic dyes and quantum dots. However, their QYs are generally low and thus need to be improved to facilitate and extend their applications. Considerable efforts have been made to improve the QYs of Ln^3+^ ion-based nanoparticles through photosensitization. These efforts include the doping of Ln^3+^ ions into host matrices or capping the nanoparticles with organic ligands. Among the Ln^3+^ ion-based nanoparticles investigated in previous studies, this review focuses on those containing Eu^3+^, Tb^3+^, and Dy^3+^ ions with red, green, and yellow emission colors, respectively. The emission intensities of Eu^3+^ and Tb^3+^ ions are stronger than those of other Ln^3+^ ions; therefore, the majority of the reported studies focused on Eu^3+^ and Tb^3+^ ion-based nanoparticles. This review discusses the principles of photosensitization, several examples of photosensitized Ln^3+^ ion-based nanoparticles, and in vitro and in vivo biomedical fluorescent imaging (FI) applications. This information provides valuable insight into the development of Ln^3+^ ion-based nanoparticles with high QYs through photosensitization, with future potential applications in biomedical FI.

## 1. Introduction

Photoluminescent (PL) trivalent lanthanide (Ln^3+^) ion-based nanoparticles have attracted considerable interest in science and technology owing to their outstanding fluorescent properties in the visible region, such as nearly constant transition energies, long emission lifetimes, atomic-like sharp transitions, large Stokes shifts, high photostability, and resistance to photobleaching [1,2,3,4,5]. These excellent fluorescent properties are highly valuable for their application in light-emitting diodes (LEDs), solar cells, biolabeling, bioimaging, and other systems [1,2,5,6,7,8,9,10]. In particular, Eu^3+^ and Tb^3+^ ion-based nanoparticles with emission colors of red and green, respectively, have been intensively investigated owing to their stronger emission intensity compared with other Ln^3+^ ion-based nanoparticles. Dy^3+^ ion-based nanoparticles (with yellow emission color) followed Eu^3+^ and Tb^3+^ ion-based nanoparticles, owing to their weaker emission intensity. In particular, the time-resolved (or delayed) detection ability of Ln^3+^ ion-based nanoparticles (owing to their long emission lifetimes in the micro-to-millisecond range), which is not available for traditional organic dyes and quantum dots (QDs) with short emission lifetimes (on the order of nanoseconds) [11], makes the Ln^3+^ ion-based nanoparticles unique fluorescent probes, enabling interference-free signal detection without background autofluorescence from solution samples, which typically occurs on a nanosecond timescale [12,13]. Note that the organic dyes have high QYs but low photostability, low resistance to photobleaching, broad emission, small Stokes shifts, and short emission lifetimes, whereas the QDs have appreciable QYs, narrow emission, high resistance to photobleaching, and good photostability, but small Stokes shifts, short emission lifetimes, and high toxicity. Despite many excellent fluorescent properties of Ln^3+^ ion-based nanoparticles, superior to those of organic dyes and QDs, their intrinsic low quantum yields (QYs) limit their applications. This is due to the forbidden 4f–4f transitions of the Ln species, which result in a low molar absorption coefficient [14,15]. Strategies involving the use of photosensitizers such as host matrices or capping organic ligands were employed to overcome this issue [16,17,18,19,20,21,22]. The performance of fluorescent probes critically depends on their QYs; therefore, the development of Ln^3+^ ion-based nanoparticles with high QYs is essential to facilitate and enhance their applications.

The fluorescent properties of Ln^3+^ ion-based nanoparticles can be significantly impaired by solvents, particularly water molecules, owing to their coordination with the Ln^3+^ ions on nanoparticle surfaces [23]. Water molecules can promote the nonradiative energy transfer from Ln^3+^ ions, reducing the radiative relaxation of Ln^3+^ ions. Such solvent-induced fluorescence quenching not only reduces the QYs, but also shortens the emission lifetimes, therefore significantly impairing the fluorescent properties of Ln^3+^ ions, limiting their applications in aqueous media. This problem can be overcome via photosensitization using organic capping ligands [18] or core–shell structures [20,21,22].

**Scheme 1:**
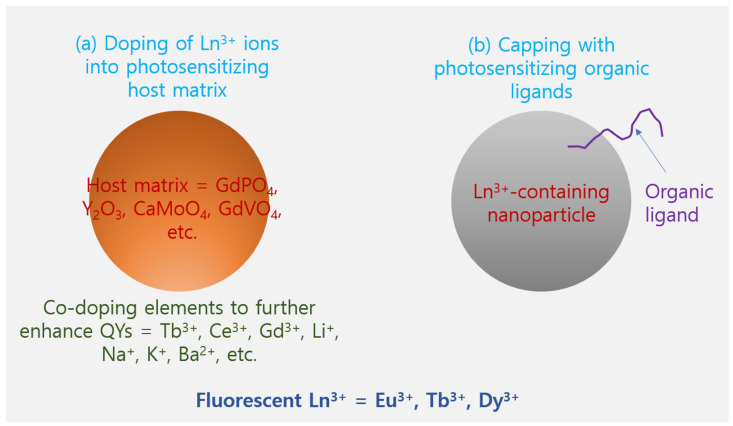
Two types of strategies to enhance QYs: (**a**) doping of Ln^3+^ ions into photosensitizing host matrix (co-doping elements further enhance QYs) and (**b**) capping of Ln^3+^-containing nanoparticles with photosensitizing organic ligands.

As shown in Scheme 1, two photosensitization strategies to improve the QYs of Ln^3+^ ion-based nanoparticles have been reported to date (Table 1) [12,17,18,20,21,24,25,26,27,28,29,30,31,32,33,34,35,36,37,38,39,40,41,42,43,44,45,46,47,48,49,50,51,52,53,54,55,56,57,58,59,60,61,62,63,64,65,66,67,68,69,70,71,72,73,74,75,76,77,78,79,80,81,82,83,84,85,86,87,88,89,90]: one involves embedding Ln^3+^ ions into photosensitizing host matrices [20,21,24,25,26,27,28,29,30,31,32,33,34,35,36,37,38,39,40,41,42,43,44,45,46,47,48,49,50,51,52,53,54,56,57,58,59,60,61,62,63,64,65,66,67,68,70,72,73,75,76,77,78,81,82,83,84,87,88,89,90] (Scheme 1a), while the other consists of capping the nanoparticles with photosensitizing organic ligands [17,18,55,69,74,79,80,85,86] (Scheme 1b). As shown in Table 1, most studies have employed host matrices. The strategies involving capping with organic ligands have been mostly applied for molecular Ln^3+^ ion complexes [91,92,93,94,95] but not for Ln^3+^ ion-based nanoparticles [17,18,55,69,74,79,80,85,86]. Recently, ultrasmall Ln_2_O_3_ nanoparticles (Ln = Eu, Tb, and Dy) grafted with poly acrylic acid (PAA) and the organic photosensitizer 2,6-pyridinedicarboxylic acid (PDA) exhibited very high absolute QYs of 87.6%, 73.6%, and 2.8% for Ln = Eu, Tb, and Dy, respectively, in aqueous media [18]. The study demonstrated the possibility of achieving high QYs in aqueous media relevant for biomedical fluorescence imaging (FI) applications. On the other hand, various host matrices such as GdPO_4_, Y_2_O_3_, CaMoO_4_, and GdVO_4_ have been used as photosensitizers [33,55,63,70,71,72,73]. In addition, the concentration of Ln^3+^ ions in the host matrices has been carefully varied to obtain optimal QYs by minimizing fluorescence quenching by Ln^3^⁺ ions, which generally takes place at high Ln^3+^ ion concentrations [96]. The secondary metal ions such as Ce^3+^ or Tb^3+^ or Gd^3+^ ions co-doped with Ln^3+^ ions in host matrices have provided further enhanced QYs by facilitating energy transfer from the host matrix to the fluorescent Ln^3+^ ions and also acting as photosensitizers [24,27,29,31,32,33,34,35,36,51,52,54,55,57,60,75,77,89]. Moreover, co-doping with Li^+^, Na^+^, K^+^, and Ba^2+^ ions led to further improved QYs by breaking the parity selection rule, modifying the local crystal field, reducing lattice strains, and altering the crystal phase [45,97,98,99].

**Figure 1:**
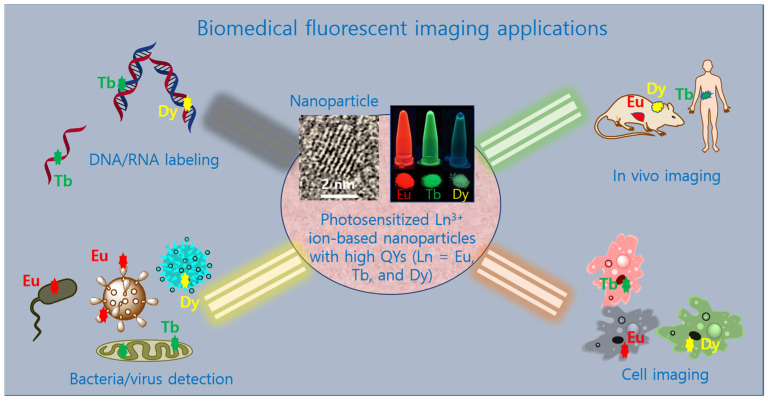
Potential application areas of photosensitized Ln^3+^ ion-based nanoparticles (Ln = Eu, Tb, and Dy) with high QYs in biomedical FI. TEM image and fluorescent samples taken from [18].

In this review, we discuss Ln^3+^ ion-based nanoparticles with high QYs through photosensitization by organic ligand capping or the doping of Ln^3+^ ions into host matrices, focusing on Ln = Eu, Tb, and Dy because they are especially useful, owing to their unique intense red, green, and yellow emissions, respectively, compared with other Ln^3+^ ion-based nanoparticles. As provided in Figure 1, the potential biomedical FI application areas of photosensitized Ln^3+^ ion-based nanoparticles with high QYs include the sensitive detection of DNA/RNA and infectious disease, such as bacteria and virus, and in vitro and in vivo imaging of normal/cancer cells and living objects, respectively. Examples of photosensitized Ln^3+^ ion-based nanoparticles and their biomedical FI applications are described. We also discuss the underlying mechanisms and the role of photosensitizers in enhancing the QYs.

## 2. QY Enhancement Strategies via Photosensitization and Corresponding Mechanism

### 2.1. QY

Ln^3+^ ions exhibit low QYs, owing to the forbidden 4f–4f electronic transitions by selection rules [18,100]. The QY is an important parameter to determine the performance of fluorescent materials, defined as the ratio between the number of emitted and absorbed photons (Equation (1)):

(1) $$QY=\frac{number\;of\;photons\;emitted}{number\;of\;photons\;absorbed}$$

A high QY indicates that a significant proportion of the absorbed photons are successfully emitted, indicating an excellent fluorescent probe. Examples of host matrices and organic ligands previously used as photosensitizers are discussed below.

### 2.2. Host Matrices as Photosensitizers

**Figure 2:**
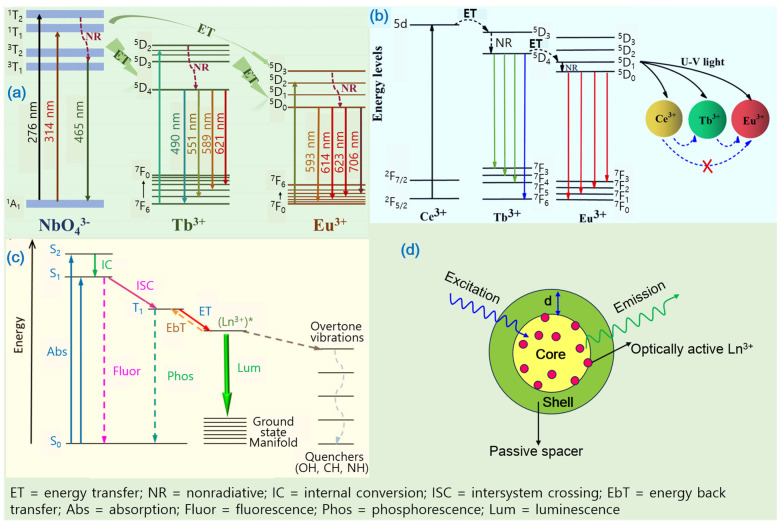
Diagrams showing energy transfer (**a**) from host matrix (i.e., NBO_4_^3−^) to Tb^3+^ and Eu^3+^ ions and from Tb^3+^ to Eu^3+^ ions [110], (**b**) from Ce^3+^ to Tb^3+^ ions and from Tb^3+^ to Eu^3+^ ions [57], and (**c**) from organic ligands to excited (Ln^3+^)* ions: the right panel shows the fluorescent quenching via nonradiative energy transfer from excited (Ln^3+^)* ions to conjugated functional groups (OH, CH, and NH) of solvent or organic molecules [112]. (**d**) Schematic diagram of core–shell structure for QY enhancement through surface passivation of the core nanoparticle with the shell.

As provided in Table 1, the photosensitization of Ln^3+^ ion-based nanoparticles mostly involves host matrices [20,21,24,25,26,27,28,29,30,31,32,33,34,35,36,37,38,39,40,41,42,43,44,45,46,47,48,49,50,51,52,53,54,56,57,58,59,60,61,62,63,64,65,66,67,68,70,72,73,75,76,77,78,81,82,83,84,87,88,89,90]. The host matrices serving as photosensitizers should possess several key features [101,102,103,104,105,106,107]: (1) low phonon (i.e., lattice vibrational) energies to minimize energy losses via nonradiative energy decay [101]; (2) the ability to host a high concentration of Ln^3+^ ions while maintaining their photosensitization performance [102]; (3) a high optical transparency to the fluorescent wavelengths of the Ln^3+^ ions [103,104]; (4) chemical and thermal stabilities after being doped with Ln^3+^ ions [105]; (5) a sufficient energy gap from Ln^3+^ ions to avoid back energy transfer to them [106]; and (6) a close lattice match with Ln^3+^ ions [107]. The trivalent La^3+^, Ce^3+^, Gd^3+^, Lu^3+^, and Y^3+^ ions exhibit similar ionic radii and can thus easily host Ln^3+^ ions. Various rare earth-based materials have been explored as host matrices, including oxides, fluorides, phosphates, borates, and vanadates [108]. In particular, fluoride-based rare earth materials are more advantageous than other host matrices owing to their lower phonon energies [108,109]. Figure 2a displays a simplified diagram showing how Ln^3+^ ions are photosensitized through the energy transfer from excited states of the host matrix to Ln^3+^ ions [110]. As shown in Figure 2a, the NBO_4_^3−^ ions of the host matrix absorb incoming photons and then transfer energy to Tb^3+^ and Eu^3+^ ions to emit photons. In addition, the Tb^3+^ ions also act as photosensitizers, transferring their energy to Eu^3+^ ions to emit photons. In the case of co-doping, Ce^3+^ ions absorb photons and transfer energy to Tb^3+^ ions (but not Eu^3+^ ions, owing to the large energy gap between the excited energy levels of Ce^3+^ and Eu^3+^ ions); then, Tb^3+^ ions transfer energy to Eu^3+^ ions to emit photons, as shown in Figure 2b [57]. For biomedical FI applications, the nanoparticles should be stable as colloids in aqueous media; therefore, they should be grafted with hydrophilic polymers such as PAA [18,24,111]. However, the polymers should not interfere with the PL of the nanoparticles.

### 2.3. Organic Ligands as Photosensitizers

Although organic ligand photosensitizers have been less studied than host matrices (Table 1), they have been proven to be highly efficient in enhancing the QYs of Ln^3+^ ion-based nanoparticles [18,19,113,114,115,116,117]. However, as organic photosensitizers are not highly hydrophilic, hydrophilic polymers such as PAA should also be grafted on the nanoparticles to obtain stable colloids in aqueous media for biomedical FI applications [18,118]. Figure 2c displays a simplified diagram showing how Ln^3+^ ions are photosensitized through energy transfer from the excited states of organic ligands [112]. As shown in Figure 2c, the photosensitizing process involves the excitation of ligands from the singlet ground state (S_0_) to several singlet excited states (S_i_, i = 1, 2, 3, …), followed by nonradiative energy decay [denoted as internal conversion (IC)] from Si (i = 2, 3, …) to S_1_, and then by another nonradiative energy decay [intersystem crossing (ISC)] to the triplet (T_1_) state. Then, nonradiative energy transfer takes place from the T_1_ state of the organic ligands to the excited states of the Ln^3+^ ions, followed by fluorescence to the lower energy states of the Ln^3+^ ions. To achieve efficient energy transfer to the Ln^3+^ ions, the energy of the T_1_ excited state of the organic ligands should be higher than the emitting levels of the Ln^3+^ ions. However, very large energy gaps between the T_1_ state of the organic ligands and the excited states of the Ln^3+^ ions will lead to ineffective energy transfer, whereas a small energy gap may lead to the back energy transfer from the excited Ln^3+^ ions to the T_1_ state of the organic ligands. According to Latva’s empirical rule, to achieve efficient photoluminescence (PL), the T_1_ excited state of the organic ligands should be 1850 to 4000 cm^−1^ higher than the emitting levels of the Ln^3+^ ions [119].

### 2.4. Surface Passivation by Thin Layer of Optically Transparent Undoped Matrix

As illustrated on the right side of Figure 2c, the fluorescent quenching by solvents, especially water, which occurs through nonradiative energy transfer between the Ln^3+^ ions and OH groups of water, significantly reduces the QYs of Ln^3+^ ion-based nanoparticles in aqueous media [18,23]. As the particle size decreases, the quenching effect becomes more severe, owing to the higher surface to volume ratios. Surface passivation of the nanoparticles was employed to mitigate this issue, by growing an optically transparent layer to form a core–shell structure (Figure 2d). This surface passivation protects the surface Ln^3+^ ions from fluorescence quenching [20,21,35]. 

## 3. Examples of Photosensitized Ln^3+^ Ion-Based Nanoparticles

### 3.1. Organic Ligand Photosensitization

**Figure 3:**
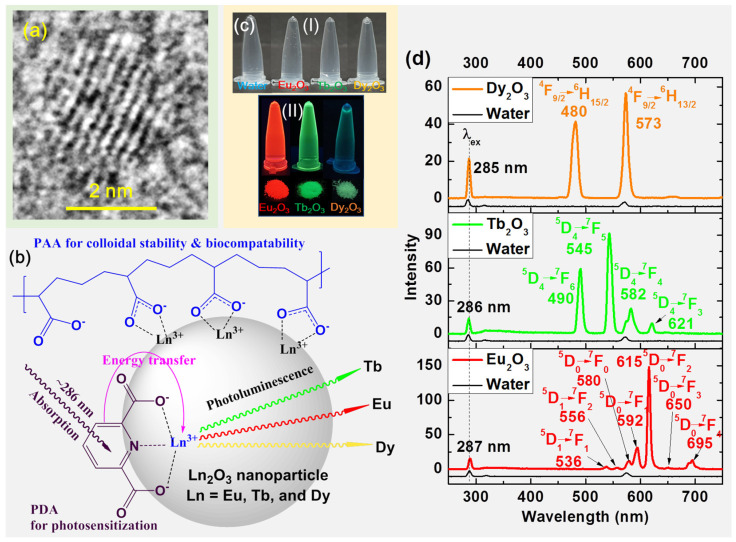
(**a**) TEM image at 2 nm scale. (**b**) Schematic illustration of bonding structure of PAA and PDA on ultrasmall Ln_2_O_3_ (Ln = Eu, Tb, or Dy) nanoparticle surface. (**c**) Photographs of water and PAA/PDA–Ln_2_O_3_ (Ln = Eu, Tb, or Dy) nanoparticle colloids in aqueous media (**I**) before and (**II**) under 254 nm UV irradiation, showing stable colloids in aqueous media along with strong red and green emissions for Ln = Eu and Tb, respectively, as well as pale-yellow emissions for Ln = Dy. (**d**) Concentration-normalized PL spectra of PAA/PDA–Ln_2_O_3_ (Ln = Eu, Tb, and Dy) nanoparticle colloids in aqueous media, with excitation wavelengths (λ_ex_s) of 287, 286, and 285 nm, respectively [18].

There has been growing interest in using organic ligands as photosensitizers to enhance the QY of Ln^3+^ ion-based nanoparticles [18,19,69,116,120,121,122]. For example, PAA-grafted ultrasmall Ln_2_O_3_ nanoparticle colloids (Ln = Eu, Tb, or Dy) with high QYs in aqueous media were prepared using PDA as a photosensitizer [18]. The ultrasmall PAA/PDA–Ln_2_O_3_ nanoparticle colloids were nearly monodispersed, with a particle diameter of ~2 nm [see transmission electron microscopy (TEM) image in Figure 3a]. The absolute QYs of the nanoparticle colloids in aqueous media were 87.6, 73.6, and 2.8% for Ln = Eu, Tb, and Dy, respectively. These values were the highest reported to date for the corresponding Ln^3+^ ion-based nanoparticles in aqueous media. Figure 3b schematically shows the bonding structure of the PAA and PDA ligands on the nanoparticle surface through their COO^−^ and N groups. Figure 3c displays photographs of water and nanoparticle colloidal solutions in aqueous media without and with 254 nm ultraviolet (UV) irradiation, respectively, showing stable colloids without precipitation and strong fluorescence in aqueous media. Moreover, Figure 3d shows the concentration-normalized PL spectra of the PAA/PDA–Ln_2_O_3_ nanoparticle colloids (Ln = Eu, Tb, and Dy) in aqueous media, with excitation wavelengths of 287, 286, and 285 nm, respectively. The emission peaks were sharp (full widths at half maximum = ~10.5 nm, similar to atomic-like transitions) and strong, owing to the PDA photosensitization. Cellular cytotoxicity assays showed that these nanoparticle colloids were nontoxic, highlighting their promising potential as fluorescent probes for biomedical FI applications.

### 3.2. Host Matrix Photosensitization

#### 3.2.1. Photosensitized Eu^3+^ Ion-Doped Nanoparticles

**Figure 4:**
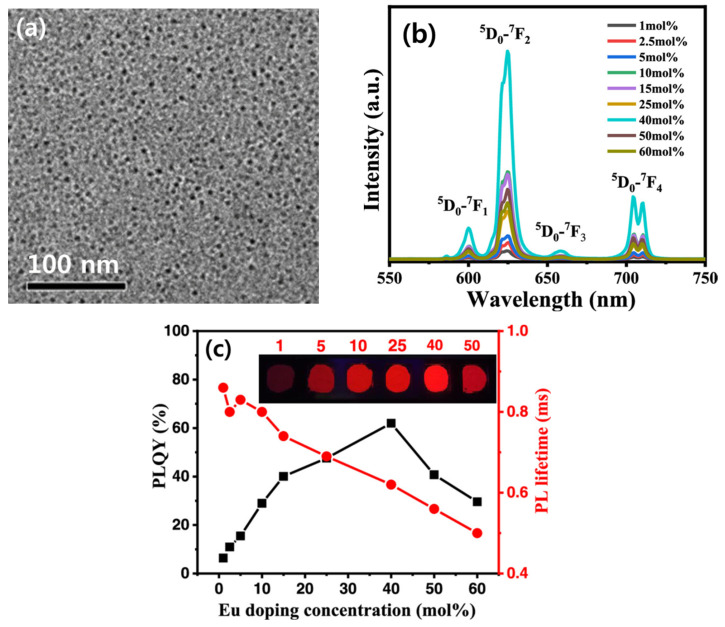
(**a**) TEM image of as-prepared Y_0.6_VO_4_:Eu_0.4_^3+^ nanoparticles at 100 nm scale. (**b**) PL spectra of Y_1−x_VO_4_:Eu_x_^3+^ nanoparticles at various Eu^3+^ ion-doping concentrations (x = 0.01, 0.025, 0.05, 0.1, 0.15, 0.25, 0.4, 0.5, and 0.6) (λ_ex_ = 310 nm). (**c**) PLQYs (left) and PL lifetimes (right) of Y_1−x_VO_4_:Eu_x_^3+^ nanoparticles as a function of Eu^3+^ ion-doping concentration; the inset shows photographs of the nanoparticle powder samples (x = 0.01, 0.05, 0.1, 0.25, 0.4, and 0.5) under 254 nm UV irradiation [70].

Previous studies investigated QYs [16,59,64,65,66,67,68,123,124,125,126,127], QY enhancement strategies [17,60,61,128,129], and biomedical FI applications of a variety of Eu^3+^ ion-doped nanoparticles [12,56,62,111,130]. The Eu^3+^ ion is a well-known red phosphor, exhibiting red emissions due to transitions from ^5^D_0_ to ^7^F_J_ (J = 0, 1, 2, 3, 4, 5, 6) states, among which the transition to J = 2 at ~613 nm is the most intense. Examples of photosensitized Eu^3+^ ion-doped nanoparticles are discussed in the following:

Li et al. synthesized ultrasmall Eu^3+^ ion-doped YVO_4_ nanoparticles [particle diameter (d) = 3.6 nm] using a ligand-assisted coprecipitation method at room temperature, as shown in the TEM image of Figure 4a [70]. The PL spectra of the Y_1−x_VO_4_:Eu_x_^3+^ nanoparticles (x = 0.01, 0.025, 0.05, 0.1, 0.15, 0.25, 0.4, 0.5, and 0.6) showed the characteristic ^5^D_0_ → ^7^F_J_ (J = 1–4) transitions of Eu^3+^ (Figure 4b). The PL intensity and QY of the Eu^3+^ ion-doped YVO_4_ nanoparticles were influenced by the Eu^3+^ ion-doping concentration. An increase in concentration initially led to an increase in QY, which reached a maximum value of 62% at 40 mol% Eu^3+^ and then decreased afterward, owing to fluorescence quenching in the presence of a large amount of Eu^3+^ ions (Figure 4c, left). In addition, the PL lifetime (τ) exhibited a nearly monotonic decrease with increasing Eu^3+^ ion-doping concentration; this decrease was due to the interaction between Eu^3+^ ions, which nearly linearly depended on the Eu^3+^ ion-doping concentration (Figure 4c, right). The inset in Figure 4c shows photographs of the nanoparticle powder samples (x = 0.01, 0.05, 0.1, 0.25, 0.4, and 0.5) under 254 nm UV irradiation, displaying an Eu^3+^ ion-doping concentration-dependent fluorescent color, with the brightest red color observed at 40 mol% of Eu^3+^. This study implies that the PL intensity and QY can be optimized by optimizing the Eu^3+^ ion-doping concentration.

**Figure 5:**
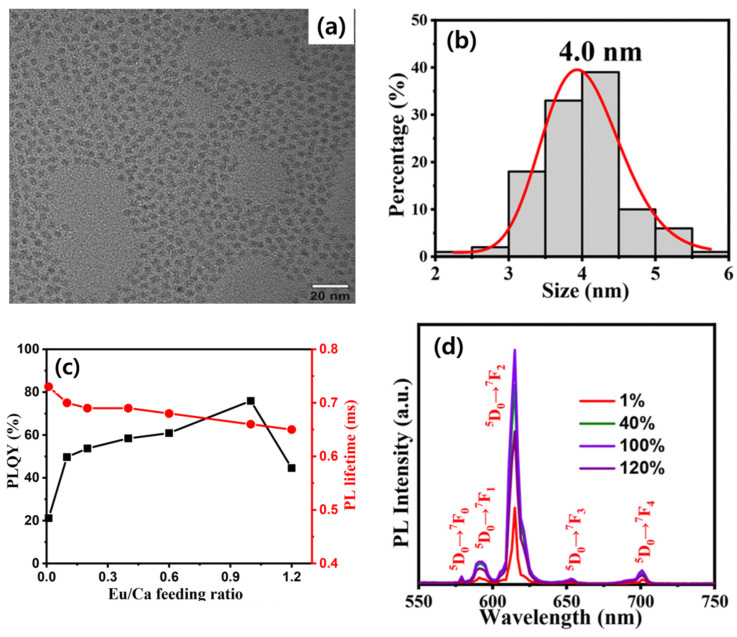
(**a**) TEM image at 20 nm scale and (**b**) particle diameter distribution of Eu^3+^ ion-doped CaMoO_4_ nanoparticles. (**c**) PLQYs (left) and PL lifetimes (right) of Eu^3+^ ion-doped CaMoO_4_ nanoparticles as a function of Eu/Ca ratio. (**d**) PL spectra of Eu^3+^ ion-doped CaMoO_4_ nanoparticles at various Eu/Ca ratios (λ_ex_ = 280 nm) [72].

Cao et al. studied the fluorescent properties and QY enhancement of Eu^3+^ ion-doped CaMoO_4_ nanoparticles [72]. As shown by the TEM image and size distribution plot in Figure 5a and Figure 5b, respectively, the nanoparticles were well dispersed in chloroform, with an average particle diameter of 4 nm. By controlling the Eu/Ca ratio, the nanoparticles were modified in order to optimize the fluorescent intensity and QY. The PL intensity first increased and then decreased with the increasing Eu/Ca ratio. This trend was attributed to the high concentration of Eu^3+^ ions in the CaMoO_4_ nanoparticles, which led to a higher probability of nonradiative decay (i.e., fluorescence quenching), owing to the energy transfer between Eu^3+^ ions at the shorter distances corresponding to high concentrations. The nanoparticles with an Eu/Ca ratio of 1:1 exhibited the highest PL intensity (Figure 5c) and PLQY (75%, Figure 5d). The PL lifetime exhibited a nearly monotonic decrease with the increasing Eu/Ca ratio (Figure 5d), owing to the stronger interaction between Eu^3+^ ions with increasing concentration.

**Figure 6:**
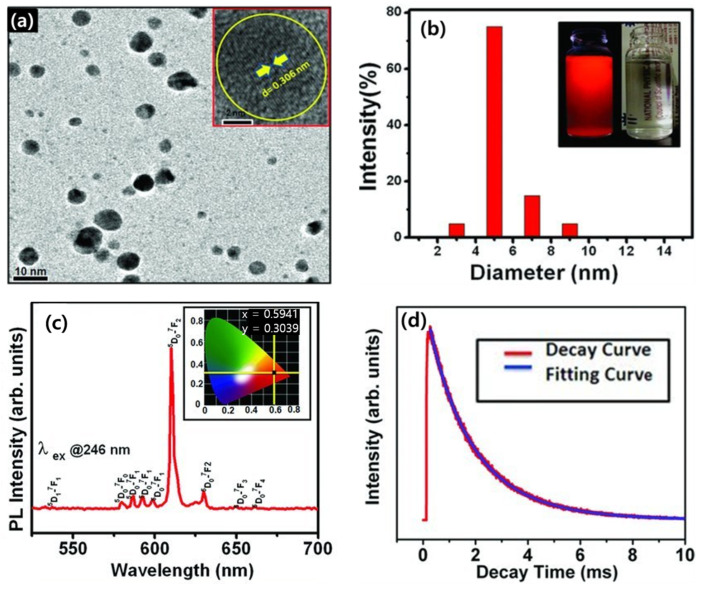
(**a**) TEM image of Y_1.9_O_3_:Eu_0.1_^3+^ nanoparticles at 10 nm scale. The inset shows a magnified TEM image at 2 nm scale, displaying lattice fringes with a lattice distance of 0.306 nm for the (222) plane. (**b**) Particle diameter distribution obtained from dynamic light scattering for Y_1.9_O_3_:Eu_0.1_^3+^ nanoparticles dispersed in deionized water. The inset shows photographs of Y_1.9_O_3_:Eu_0.1_^3+^ nanoparticle colloids in aqueous media with (left) and without (right) 254 nm UV irradiation. (**c**) PL spectrum of Y_1.9_O_3_:Eu_0.1_^3+^ nanoparticles recorded using 246 nm excitation. The inset shows the CIE color coordinates of red emission (x = 0.5941, y = 0.3039). (**d**) Time-resolved fluorescent spectrum and double-exponential fitting using lifetime parameters (τ_1_ and τ_2_) [73].

Gupta et al. reported the strong emission properties of Eu^3+^ ion-doped yttrium oxide nanoparticles (Y_1.9_O_3_:Eu_0.1_^3+^) [73]. The nanoparticles exhibited an average particle diameter of 6 nm, as shown in the TEM image and diameter distribution plot (Figure 6a and Figure 6b, respectively). The insets in Figure 6b show photographs of Y_1.9_O_3_:Eu_0.1_^3+^ nanoparticle colloids in aqueous media with (left) and without (right) 254 nm UV irradiation, showing strong red emissions. The QY of the nanoparticles was greater than 90%, confirming their high brightness and hypersensitive intense red emissions at 610 nm (^5^D_0_ → ^7^F_2_) and room temperature, as shown in the PL spectrum (Figure 6c). The inset in Figure 6c shows the Commission International de l’Eclairage (CIE) (1931) color coordinates of red emissions (x = 0.5941, y = 0.3039), which are close to the National Television System Committee (NTSC) standard for red phosphors (x = 0.67, y = 0.33). Moreover, the lifetimes obtained from the double-exponential fitting of the time-resolved PL spectrum for the ^5^D_0_ → ^7^F_2_ transition revealed τ_1_ and τ_2_ values of 1.068 and 2.097 ms, respectively (Figure 6d). The average lifetime for the transition was calculated to be τ_av_ ≈ 1.99 ms, indicating the suitability of the nanoparticles for time-resolved biomedical FI applications, enabling background autofluorescence-free signal detection from sample solutions. This time-resolved imaging can enhance the signal-to-noise ratio, enabling signal detection at very low concentrations (at the picomolar level) [131], which is extremely useful for the early detection of diseases [132].

#### 3.2.2. Photosensitized Tb^3+^ Ion-Doped Nanoparticles

Tb is another Ln whose emission intensity is similar to that of Eu but shows a green color, which is useful for various applications, including bioimaging [13,133,134,135], lighting, and displays [78,136,137]. The characteristic sharp emission peaks include ^5^D_4_ → ^7^F_J_ (J = 6, 5, 4, 3) transitions, among which the ^5^D_4_ → ^7^F_5_ one at 545 nm is the most intense. The QYs of Tb^3+^ ion-based nanoparticles were enhanced using photosensitizers such as organic ligands [79,80,117,138,139,140,141,142,143] or inorganic host matrices [81,82,83,144]; some examples are discussed below.

**Figure 7:**
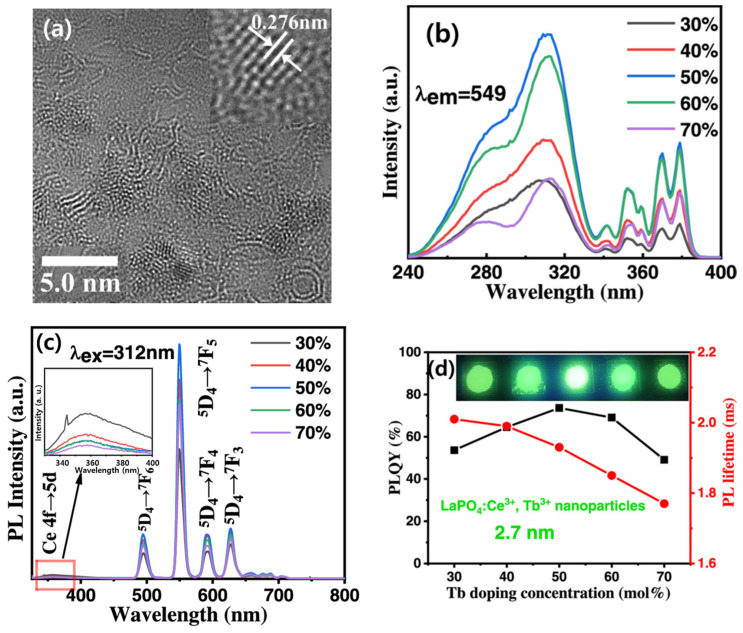
(**a**) TEM image of as-prepared La_0.4_PO_4_:Ce_0.1_^3+^,Tb_0.5_^3+^ nanoparticles at 5.0 nm scale. The inset shows a magnified TEM image, displaying the lattice distance of 0.276 nm for the (102) plane. (**b**) Excitation and (**c**) PL spectra of La_1−0.1−x_PO_4_:Ce_0.1_^3+^,Tb_x_^3+^ nanoparticles with different Tb^3+^ ion-doping concentrations (i.e., x = 0.3, 0.4, 0.5, 0.6, and 0.7). The inset in PL spectra shows magnified PL spectra between 320 and 400 nm. (**d**) PLQYs (left) and PL lifetimes (right) of La_1−0.1−x_PO_4_:Ce_0.1_^3+^,Tb_x_^3+^ nanoparticles as a function of Tb^3+^ ion-doping concentrations (i.e., x = 0.3, 0.4, 0.5, 0.6, and 0.7). The inset shows photographs of La_1−0.1−x_PO_4_:Ce_0.1_^3+^,Tb_x_^3+^ nanoparticles under 254 nm UV irradiation [33].

The QYs of Tb^3+^ ions can be enhanced through co-doping with Ce^3+^ ions, which is attributed to energy transfer from Ce^3+^ to Tb^3+^ ions [32,34,71,84,145,146]. For example, Ni et al. synthesized highly luminescent LaPO_4_:Ce^3+^,Tb^3+^ nanoparticles with a particle diameter of 2.7 nm, as shown in the TEM image of Figure 7a [33]. The excitation spectra in Figure 7b show a wide band within the 240–330 nm range peaking at ~312 nm, associated with the 4f → 5d transitions of Ce^3+^ ions, along with several sharp excitation peaks in the 330–400 nm range, corresponding to the weak 4f–4f transitions of Tb^3+^ ions, indicating a significant contribution of Ce^3+^ ions to the energy transfer to Tb^3+^ ions. The nanoparticles mainly show four PL peaks at 495, 549, 593, and 627 nm, corresponding to the ^5^D_4_ → ^7^F_J_ (J = 3, 4, 5, and 6) transitions of Tb^3+^ ions, respectively (Figure 7c). The most intense peak in the PL spectrum originated from the ^5^D_4_ → ^7^F_5_ transition at 545 nm. Additionally, a very weak and broad PL peak in the range of 320 to 400 nm arose from the 5d–4f transitions of Ce^3+^ ions (inset in Figure 7c). The Tb^3+^ ion-concentration-dependent intensities in excitation (Figure 7b) and PL (Figure 7c) spectra confirmed the energy transfer from Ce^3+^ to Tb^3+^ ions. This energy transfer was further investigated by estimating the PLQYs (Figure 7d) by increasing the Tb^3+^ ion concentration from 30 to 70% at a fixed Ce^3+^ ion concentration. As shown in the inset of Figure 7c, as the Tb^3+^ ion concentration increased, the emission intensity of Ce^3+^ ions decreased, owing to the energy transfer from Ce^3+^ to Tb^3+^ ions. The nanoparticles exhibited a remarkable PLQY of 74% and the brightest emission color (the inset photograph) at the optimal 50% Tb^3+^ ion content (Figure 7d). The PL lifetimes ranged from 1.8 to 2.0 ms, which is extremely useful for time-resolved signal detection (i.e., background autofluorescence-free signal detection) in biological samples. The τ values nearly monotonically decreased with the increasing Tb^3+^ ion concentration, owing to the interaction between Tb^3+^ ions, similar to that observed in Eu^3+^ ion-doped nanoparticles [70,73].

**Figure 8:**
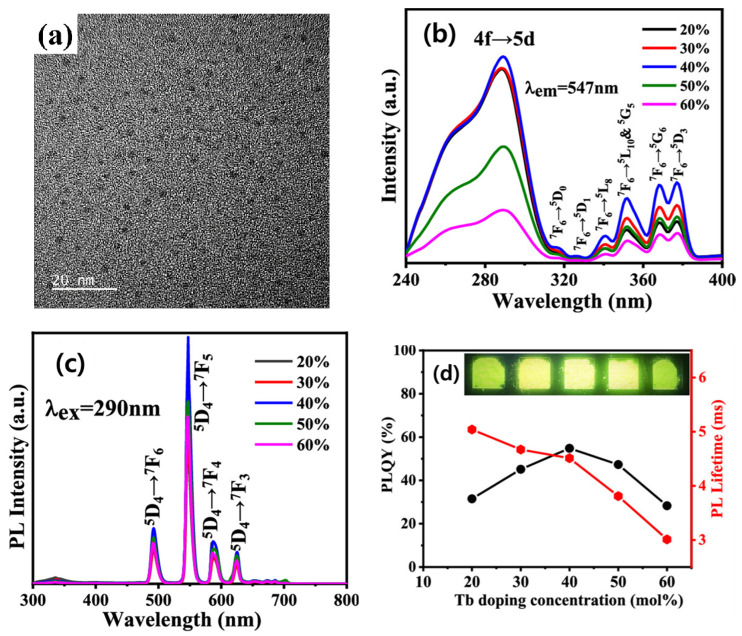
(**a**) TEM image of NaY_0. 4_F_4:_Ce^3+^_0.2_,Tb^3+^_0.4_ nanoparticles at 20 nm scale. (**b**) Excitation and (**c**) PL spectra of NaY_1−0.2−x_F_4_:Ce^3+^_0.2_,Tb^3+^_x_ nanoparticles (x = 0.2, 0.3, 0.4, 0.5, and 0.6). (**d**) PLQYs (left) and PL lifetimes (right) of NaY_1−0.2−x_F_4_:Ce^3+^_0.2_,Tb^3+^_x_ nanoparticles (x = 0.2, 0.3, 0.4, 0.5, and 0.6); the inset shows photographs of NaY_1−0.2−x_F_4_:Ce^3+^_0.2_,Tb^3+^_x_ nanoparticles (x = 0.2, 0.3, 0.4, 0.5, and 0.6) under 254 nm UV irradiation [36].

As another example of co-doping, Wei et al. synthesized NaYF_4_:Ce^3+^,Tb^3+^ nanoparticles (co-doping ions = Ce^3+^, Tb^3+^) with a high PLQY of 54.8% [36]. The TEM image in Figure 8a confirms the presence of well-dispersed nanoparticles with an average particle diameter of 2.7 nm. The excitation spectra of NaY_1−0.2−x_F_4_:Ce^3+^_0.2_,Tb^3+^_x_ nanoparticles were recorded at various Tb^3+^ ion-doping concentrations (x = 0.2, 0.3, 0.4, 0.5, and 0.6) (Figure 8b), exhibiting an optimal concentration of x = 0.4 (or 40%). The spectra displayed a strong and broad band from 240 to 320 nm with a peak at ~290 nm originating from Ce^3+^ ions, along with weak and sharp peaks between 300 and 400 nm associated with Tb^3+^ ions, indicating a high contribution of Ce^3+^ ions to the energy transfer to Tb^3+^ ions. Figure 8c shows the PL spectra at various Tb^3+^ ion-doping concentrations. The PL spectra exhibit several distinct peaks originating from Tb^3+^ ions, with the main peak at 547 nm. The optimal Tb^3+^ ion-doping concentration of 40% maximized the PL intensity and PLQY (Figure 8c and Figure 8d, respectively), whereas higher concentrations led to a reduced PL intensity and PLQY, due to the fluorescent quenching by the large amount of Tb^3+^ ions (Figure 8d). As shown in the photographs displayed in the inset of Figure 8d, the NaY_1−0.2−x_F_4_:Ce^3+^_0.2_,Tb^3+^_x_ nanoparticles exhibited a Tb^3+^ ion-doping concentration-dependent PL brightness, with the highest value obtained at x = 0.4. In addition, the fluorescence lifetime decreased with the increasing Tb^3+^ ion-doping concentration, owing to the increased interaction between Tb^3+^ ions. This study demonstrates the importance that the QY can be further enhanced by controlling the co-doping ion concentration.

**Figure 9:**
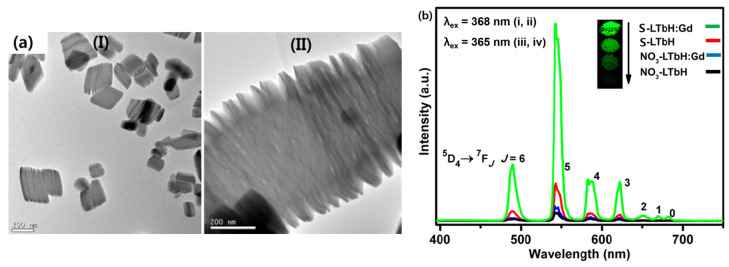
(**a**) (**I**) TEM image and (**II**) magnified TEM image of S-LTbH:Gd at 200 nm scales. (**b**) PL spectra of (i) NO_3_-LTbH, (ii) NO_3_-LTbH: Gd, (iii) S-LTbH, and (iv) S-LTbH: Gd [85]. The inset shows photographs of samples under 254 nm UV irradiation.

Liu et al. modified Gd^3+^ ion-co-doped layered terbium hydroxide (NO_3_-LTbH:Gd) nanomaterials with a photosensitizer [S = 2,2′-(((2,4,6-trimethyl-1,3-phenylene)bis(methylene))bis(oxy))dibenzoic acid] to obtain S-LTbH:Gd with enhanced fluorescent properties [85]. This nanomaterial exhibited a significant increase in QY (33%) compared to NO_3_-LTbH:Gd (QY = 23%), owing to the synergistic effect from Gd^3+^ and the intercalated photosensitizer S. As shown in the TEM images (Figure 9a(I,II)), the S-LTbH:Gd nanomaterials exhibited a well-defined layered structure with S intercalated between sheets. Figure 9b shows the PL spectra of NO_3_-LTbH, NO_3_-LTbH:Gd, S-LTbH, and S-LTbH:Gd with excitations at 368 and 365 nm, displaying transitions from the excited Tb^3+^ ions to various lower energy states: ^5^D_4_ → ^7^F_J_ (J = 6, 5, 4, 3, 2, 1, 0). The PL intensity (I) followed the order I (NO_3_-LTbH, no photosensitizer) < I (NO_3_-LTbH:Gd, photosensitization effect by Gd) < I (S-LTbH, photosensitization effect by S) < I (S-LTbH:Gd, synergistic photosensitization effect by Gd and S), demonstrating the synergistic photosensitization effect by the intercalated S and co-doped Gd^3+^ ions. The photograph of these samples under 254 nm UV irradiation (inset of Figure 9b) displayed PL brightness consistent with their PL intensity observed.

#### 3.2.3. Photosensitized Dy^3+^ Ion-Doped Nanoparticles

In comparison to Eu^3+^ and Tb^3+^ ion-doped nanoparticles, fewer reports have been published on Dy^3+^ ion-doped nanoparticles, primarily owing to their weaker PL intensity. The Dy^3+^ ion exhibits three emissions in the blue (480 nm, weak intensity), yellow (573 nm, moderate intensity), and red (660 nm, very weak intensity) regions, attributed to ^4^F_9/2_ → ^6^H_J_ electronic transitions (J = 15/2, 13/2, and 11/2, respectively); however, these emission intensities can be improved via photosensitization [42,147,148]. In addition, Dy^3+^ ion-doped nanoparticles have attracted considerable interest because of their potential as white light phosphors [41,46,149,150,151]. In general, the emission of white light can be achieved by combining three colors: red (R), green (G), and blue (B), denoted as RGB; however, it can also be obtained by combining blue and yellow [152]. The yellow and blue emissions correspond to host-sensitive electric dipole and host-insensitive magnetic dipole transitions, respectively [153,154,155]. Therefore, by adjusting the intensity ratio of yellow to blue, the Dy^3+^ ion-doped nanoparticles can provide white light fluorescence.

**Figure 10:**
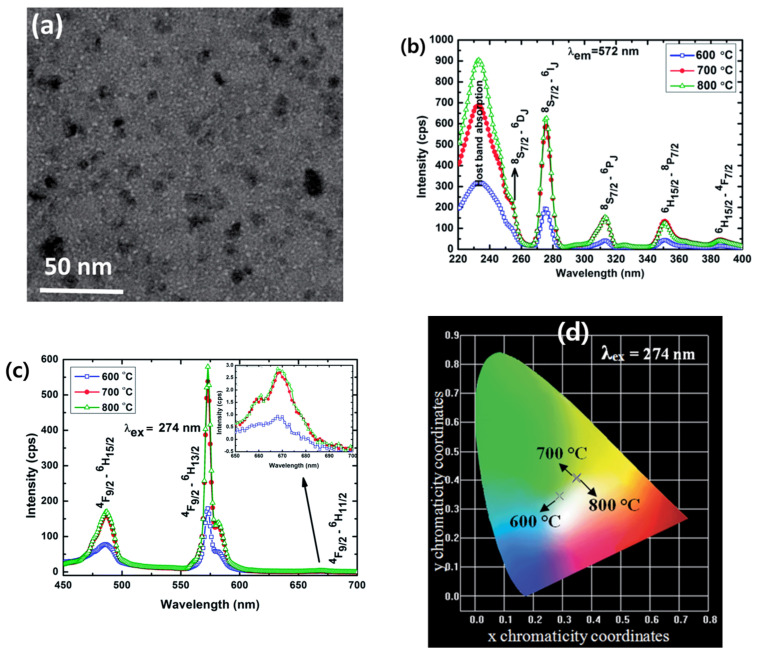
(**a**) TEM image of Gd_2_O_3_:Dy^3+^ nanoparticles annealed at 600 °C at 50 nm scale. (**b**) Excitation spectra of Gd_2_O_3_:Dy^3+^ nanoparticles prepared at annealing temperatures of 600, 700, and 800 °C with a monitoring emission wavelength of 572 nm. (**c**) PL spectra of Gd_2_O_3_:Dy^3+^ nanoparticles prepared at different annealing temperatures at an excitation wavelength of 274 nm. The inset shows magnified PL spectra between 650 and 700 nm. (**d**) CIE plot of Gd_2_O_3_:Dy^3+^ nanoparticles prepared at different annealing temperatures (600, 700, and 800 °C) [46].

Selvalakshmi et al. prepared multicolor-emitting Gd_2_O_3_:Dy^3+^ nanoparticles [46]. Multicolor PL in Gd_2_O_3_:Dy^3+^ nanoparticles was achieved by adjusting the intensity of three emission colors (i.e., yellow, blue, and weak red). The TEM image in Figure 10a shows that the diameter of the nanoparticles ranged from 9 to 16 nm. The excitation spectra of the nanoparticles that were annealed at 600, 700, and 800 °C at the monitoring emission wavelength of 575 nm show that the peak intensity increased with increasing annealing temperature from 600 to 800 °C, owing to the improved crystallinity at higher temperatures (Figure 10b); five peaks were observed at 230 nm (host absorption band), 256 nm (Gd^3+^, ^8^S_7/2_ → ^6^D_J_), 274 nm (Gd^3+^, ^8^S_7/2_ → ^6^I_J_), 313 nm (Gd^3+^, ^8^S_7/2_ → ^6^P_J_), 350 nm (Dy^3+^, ^6^H_15/2_ → ^6^P_7/2_), and 384 nm (Dy^3+^, ^6^H_15/2_ → ^4^F_7/2_). Figure 10c shows the PL spectra of the nanoparticles obtained by exciting Gd^3+^ ions as photosensitizers at 274 nm. The PL spectra exhibited a strong yellow emission at 572 nm (^4^F_9/2_ → ^6^H_13/2_), an appreciable blue emission at 486 nm (^4^F_9/2_ → ^6^H_15/2_), and a very weak red emission at 669 nm (^4^F_9/2_ → ^6^H_11/2_, inset in Figure 10c), demonstrating the multiple color emissions of Dy^3+^ ions. As shown in Figure 10c, similar PL intensities of blue and yellow emissions were observed at 600 °C; however, the blue emission was suppressed at higher annealing temperatures, while the yellow emission was enhanced, leading to a color shift in the CIE point towards the yellow region, as shown in Figure 10d. The appreciable Qys observed can be attributed to the energy transfer from Gd^3+^ to Dy^3+^ ions, which increased to 18.52% (600 °C), 21.277% (600 °C), and 21.602% (600 °C) with increasing annealing temperatures, owing to the enhanced crystallinity at higher temperatures as observed in emission and PL spectra. This study suggests that color tuning and white light emission using only Dy^3+^ ion-doped nanoparticles can be achieved through temperature control, which might be useful for temperature monitoring or temperature-sensitive detection.

**Figure 11:**
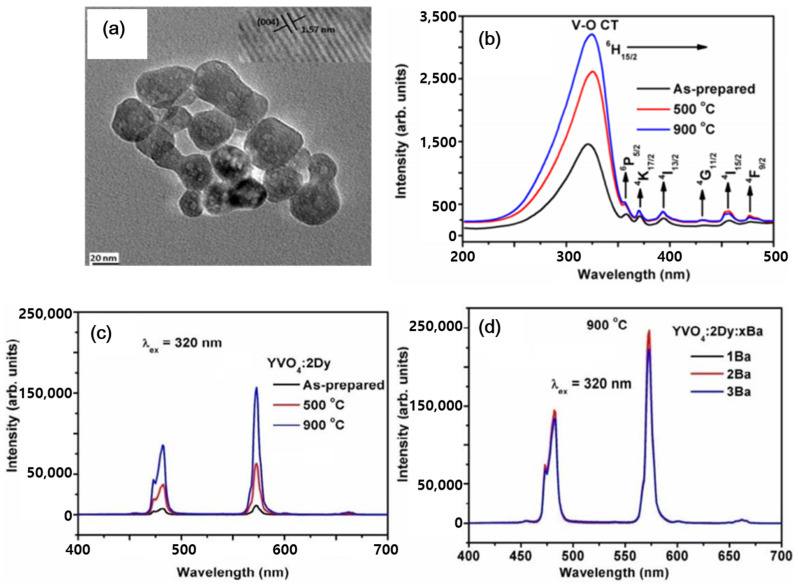
(**a**) TEM image of YVO_4_:2Dy nanoparticles annealed at 500 °C at 20 nm scale: the inset displays a magnified TEM image, showing the lattice distance of 0.157 nm for the (004) plane. (**b**) Excitation spectra of YVO_4_:2Dy nanoparticles monitored at 574 nm without and with annealing at temperatures of 500 and 900 °C. (**c**) PL spectra of YVO_4_:2Dy nanoparticles at 320 nm excitation wavelength without and with annealing at temperatures of 500 and 900 °C. (**d**) PL spectra at 320 nm excitation of Ba^2+^ ion co-doped YVO_4_:2Dy nanoparticles with different Ba^2+^ ion concentrations annealed at 900 °C [45].

Priyobarta et al. prepared Ba^2+^ ion co-doped YVO_4_:2Dy nanoparticles with a spherical shape, whose diameter typically ranged from 20 to 45 nm, as shown in the TEM image of Figure 11a [45]. As shown in Figure 11b, the excitation spectra of the YVO_4_:2Dy nanoparticles displayed broad absorption peaks between 250 and 350 nm, associated with the V–O charge transfer (CT) and weak sharp peaks associated with transitions from ^6^H_15/2_ to ^6^P_5/2_, ^4^K_17/2_, ^4^I_13/2_, ^4^G_11/2_, ^4^I_15/2_, and ^4^F_9/2_ states of Dy^3+^ ions, demonstrating the high contribution of the YVO_4_ matrix to photosensitization. The peak intensities in the excitation and PL spectra (Figure 11b,c, respectively) increased with increasing annealing temperature, owing to the enhanced crystallinity of the nanoparticles at higher annealing temperatures and the increased reduction in surface –OH bonds. The PL spectra show peaks originating from Dy^3+^ ions at 483 nm (blue) and 574 nm (yellow), along with a very weak peak at 655 nm (red). The PL intensity also depended on the co-doping Ba^2+^ ion concentration, owing to the changing lattice strain (Figure 11d). The optimal concentration of Ba^2+^ ions of the annealed nanoparticles at 900 °C was determined to be 2 at. %, owing to the reduced lattice strain, while higher concentrations (3 at. %) resulted in a decreased intensity, as shown in Figure 11d. The QY of the YVO4:2Dy nanoparticles was 29% and increased to 47% for the YVO_4_:2Dy:2Ba nanoparticles, demonstrating the boosting effect of Ba^2+^ ion co-doping on the QY.

As the particle size increases, the surface to volume ratio and thus the amount of surface Ln^3+^ ions decreases, leading to an enhanced PL intensity, owing to the reduced surface fluorescence quenching at larger particle sizes. Li et al. demonstrated this effect by increasing the diameter (150, 250, and 300 nm) of (Gd_1−x_Dy_x_)_2_O_3_ nanoparticles and observing the enhanced PL intensity of larger nanoparticles [40].

#### 3.2.4. Photosensitized Eu^3+^/Tb^3+^ Ion-Based Nanoparticles

Upon excitation, the Eu^3+^ and Tb^3+^ ions emit red and green light, respectively. When both ions are co-doped in the same host matrix, both blue and red colors can be observed; in addition, energy transfer from Tb^3+^ to Eu^3+^ ions can occur, leading to an increase in the QY for the red emission as well as in the overall QY of the nanoparticles [156,157,158,159]. Furthermore, by adjusting the molar ratio of Eu^3+^ to Tb^3+^ ions, the emission color can be adjusted between green and red. This tunability can be very useful for applications such as display technologies and lighting systems [156,160].

**Figure 12:**
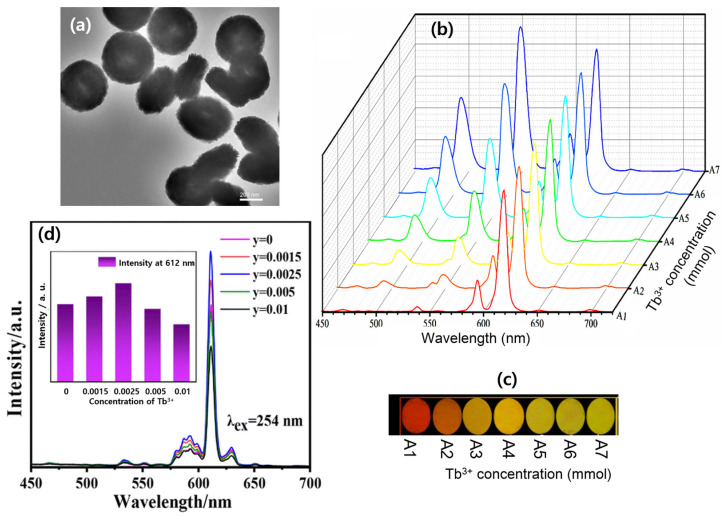
(**a**) TEM image of GdVO_4_:Eu^3+^/Tb(BA)_3_Phen sub-microflowers at 200 nm scale. (**b**) PL spectra of GdVO_4_:Eu^3+^/Tb(BA)_3_Phen at various Tb^3+^ ion concentrations (A1 = 0, A2 = 0.1, A3 = 0.2, A4 = 0.4, A5 = 0.6, A6 = 0.8, and A7 = 1.0 mmol) under 290 nm excitation. (**c**) Digital camera photographs of fluorescent samples under 254 nm UV irradiation [55]. (**d**) PL spectra of Y_1.9−y_O_3_:Eu^3+^_0.1_,Tb^3+^_y_ nanoparticles (y = 0.0015, 0.0025, 0.005, and 0.01). The inset shows the variation in PL intensity with changing y values [57].

Song et al. prepared monodisperse multicolor Tb-co-doped GdVO_4_:Eu^3+^ sub-microflowers (Figure 12a) [55]. They achieved tunable color emission by combining GdVO_4_:Eu^3+^ with Tb(BA)_3_Phen (BA = benzoate and Phen = 1,10-phenanthroline) to obtain GdVO_4_: Eu^3+^/Tb (BA)_3_Phen sub-microflowers. Figure 12b displays the PL spectra of the GdVO_4_:Eu^3+^/Tb(BA)_3_Phen material at various Tb^3+^ ion concentrations (0, 0.1, 0.2, 0.4, 0.6, 0.8, and 1.0 mmol Tb^3+^) under 290 nm excitation, showing new peaks from Tb^3+^ ions and the changes in intensity with varying Tb^3+^ ion concentrations. The characteristic PL peaks of the Eu^3+^ ions at 538, 559, 592, 619, 652, and 700 nm appeared, corresponding to the ^5^D_1_ → ^7^F_J_ (J = 1,2) and ^5^D_0_ → ^7^F_J_ (J = 1, 2, 3, and 4) transitions of the Eu^3+^ ions, respectively. With the introduction of Tb^3+^ ions by Tb(BA)_3_Phen on the surface, the additional PL peaks were observed at 492, 547, and 589 nm, which are attributed to the ^5^D_4_ → ^7^F_J_ (J = 6, 5, 4) transitions of the Tb^3+^ ions, respectively. The overall QYs (from both Eu and Tb) of the GdVO_4_: Eu^3+^/Tb(BA)_3_Phen material under 290 nm excitation increased with the increasing Tb^3+^ ion concentration, with values of 10.6, 17.3, 28.5, 35.9, 42.5, 50.7, and 62.9% at 0, 0.1, 0.2, 0.4, 0.6, 0.8, and 1.0 mmol Tb^3+^, respectively, reflecting the energy transfer from Tb^3+^ to Eu^3+^ ions. Figure 12c shows the tunable emission color, illustrating the color change from orange-red to yellow-green by adjusting the Tb^3+^ amount from 0.1 to 1.0 mmol.

Liu et al. showed that Tb^3+^ ion doping in Y_2_O_3_:Eu^3+^,Tb^3+^ nanoparticles (d = ~2.9 μm) enhanced the fluorescence performance of Eu^3+^ ions through energy transfer from Tb^3+^ ions [57]. The PL spectra of Y_1.9−y_O_3_:Eu^3+^0.1,Tb_y_^3+^ nanoparticles were measured under 254 nm excitation (Figure 12d). When the doping concentration of Tb^3+^ ions was y = 0.0025, the PL intensity of Eu^3+^ ions at 612 nm was the highest, and the QY of the powder sample with this composition was estimated to be 88.2%.

### 3.3. QY Enhancement Through Surface Passivation: Core–Shell Structure

Owing to their high surface to volume ratio, nanoparticles can suffer from surface nonradiative fluorescence quenching. However, a large amount of fluorescent Ln^3+^ ions (Ln = Eu, Tb, and Dy) on the nanoparticle surface can be protected from fluorescence quenching using a core–shell structure, leading to improved QYs and emission lifetimes. Kömpe and co-workers synthesized Tb^3+^ ion-doped CePO_4_ core nanoparticles coated with a LaPO_4_ shell [21]. The coating shell led to a significant enhancement in the QY of Tb^3+^ ions from 43 to 70%. Fang et al. developed CePO_4_:Tb nanowires coated with a LaPO_4_ shell, which led to a significant enhancement in QY from 38 to 74% [20]. The QYs increased from 47 to 83% if the Ce^3+^ emission was included. These QYs are comparable to those of the corresponding bulk material, i.e., 86% for Tb^3+^ emission and 93% including the Ce^3+^ emission. Stouwdam et al. prepared LaF_3_/Ce,Tb nanoparticles with LaF_3_ serving as shell in a core–shell structure [35]. The QY of the LaF_3_/Ce,Tb nanoparticles was significantly enhanced from 24 to 54% using the LaF_3_ shell. These results confirm the importance of surface passivation to protect surface Ln^3+^ ions from fluorescence quenching.

## 4. In Vitro and In Vivo Biomedical FI Applications

FI is a very useful technique in biomedicine, owing to its high sensitivity, non-invasiveness, and cost-effectiveness [161]. A variety of Ln^3+^ ion-based nanoparticles (Ln = Eu, Tb, and Dy) have been synthesized and employed in biomedical FI applications [162,163,164,165]. In particular, those including Eu^3+^ and Tb^3+^ ions have attracted much attention as FI agents, owing to their stronger fluorescent intensities compared with those containing Dy^3+^ ions. The photosensitized Ln^3+^ ion-based nanoparticles possess high QYs and long emission lifetimes. Their biocompatibility and efficacy as FI agents in biological systems has been demonstrated in various in vitro and in vivo applications, as discussed below.

### 4.1. In Vitro Applications

**Figure 13:**
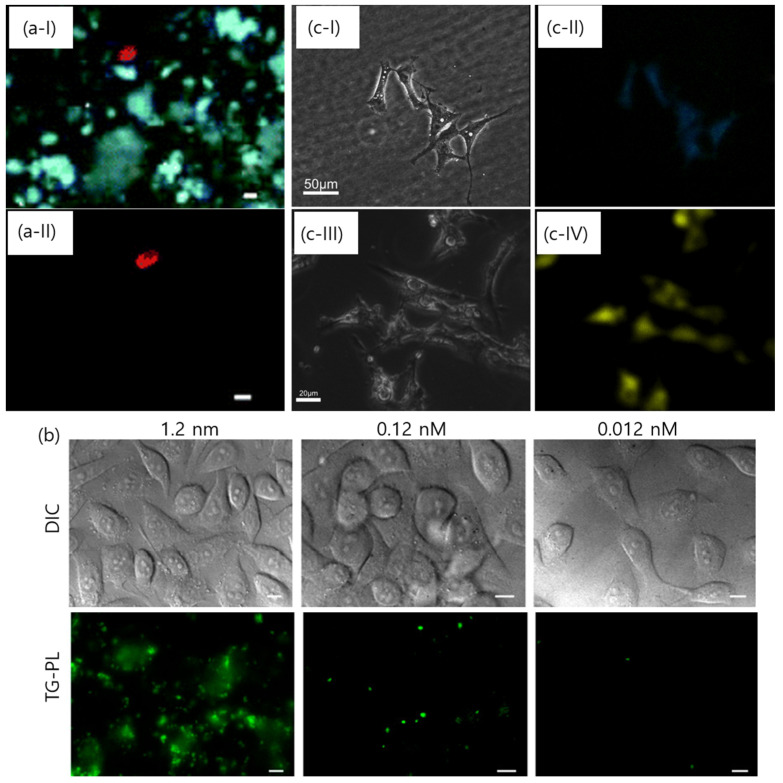
Fluorescence images of Giardia lamblia stained with SA-labeled nanoparticles (**a-I**) without and (**a-II**) with time delay (100 μs). The excitation wavelength was 380–420 nm. Scale bars: 10 µm [12]. (**b**) TG fluorescence microscopy images of HeLa cells incubated with photosensitized Tb-NPs for 24 h at different concentrations (shown on the top of Figure 13). The time delay was 10 μs. Scale bars: 10 μm [13]. DIC: differential interference contrast imaging mode in optical microscope. (**c**) In vitro FI of CNE2 cells incubated with Gd_2_O_3_:1%Dy^3+^ nanoparticles: (**c-I**,**c-III**) bright field images and (**c-II**,**c-IV**) fluorescence images captured using blue and yellow filters, respectively. Scale bars: 50 μm for (**c-I**,**c-II**), and 20 μm for (**c-III**,**c-IV**) [166].

Wu et al. developed europium nanoparticles (d = 30–40 nm) with a high QY of 66% and a long fluorescence lifetime of 389 μs, making them suitable for time-resolved FI applications [12]. They labeled these nanoparticles with streptavidin (SA) for targeting and applied them to detect Giardia lamblia (a pathogen found in environmental water samples). Strong red fluorescent signals were detected from the Giardia lamblia surface, without interference from short-lived background autofluorescence: i.e., the sky-blue autofluorescence from the sample (Figure 13a-I) disappeared with a 100 μs time delay (Figure 13a-II).

Cardoso Dos Santos et al. explored the intracellular imaging of live Henrietta Lacks (HeLa) cells without autofluorescence interference from biological samples using La_0.9_Tb_0.1_F_3_ nanoparticles (Tb-NPs) grafted with 3,3′-((butane-1,4-diylbis(azanediyl))bis(carbonyl))bis(2-hydroxy-benzoic acid) as a photosensitizer [13]. The HeLa cells were incubated with Tb-NPs at concentrations ranging from 0.012 to 1.2 nM for 24 h. As shown in Figure 13b, at the highest concentration of 1.2 nM, the nanoparticles exhibited bright PL primarily located inside the cells in time-gated (TG)-PL mode (10 μs time delay), achieving a signal-to-noise ratio of up to 40. The nanoparticles could still be visualized even at the lowest concentration of 0.012 nM, due to the absence of autofluorescence in TG-PL mode, achieving a signal-to-noise ratio of up to five.

Deng et al. applied Gd_2_O_3_:Dy^3+^ nanoparticles (d = 22.8 nm) to dual-color imaging of CNE2 cells (an epithelial cell line established from a poorly differentiated nasopharyngeal carcinoma), using the blue and yellow color emissions of Dy^3+^ ions (Figure 13c-I–c-IV) [166]. Fluorescence images acquired using filters (BA470/40 for blue and BA585/40 for yellow) revealed both blue and yellow fluorescence from the nanoparticles within the cells, as shown in Figure 13c-II,c-IV, respectively. The ability of the nanoparticles to exhibit both blue and yellow fluorescence within the cells highlights their value for dual-color FI in live cells.

### 4.2. In Vivo Applications

**Figure 14:**
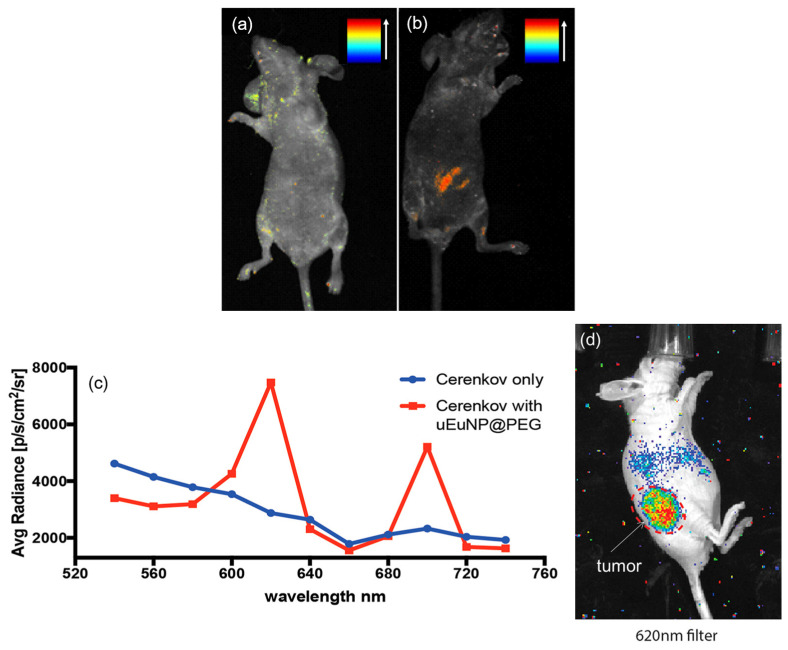
In vivo fluorescence imaging of HAP:Eu/Gd (2:1.5) nanosheets in BALB/c-nu mice: (**a**) before and (**b**) 10 min after IP injection, displaying a distinct fluorescent signal (red-orange) in enterocoelia. The arrows indicate signal intensity enhancement [167]. In vivo tumor imaging of ^89^Zr-uEuNP@PET on CT26 tumor-bearing mice: (**c**) 48 h tumor site ROI measurement spectrum vs. the regular Cerenkov spectrum and (**d**) 48 h Cerenkov imaging postinjection under 620 nm filter [164].

Less in vivo studies have been reported compared with in vitro ones. Xie et al. employed Eu/Gd co-doped hydroxyapatite [Ca_10_(PO_4_)_6_(OH)_2_, HAP] nanosheets (a plate-like nanomaterial with a 11.0 ± 2.1 nm thickness and 83.8 ± 27.3 nm size in the plane direction) for in vivo FI [167]. This HAP nanosheet matrix, serving as a photosensitizer, incorporated Eu^3+^ ions as emitters and Gd^3+^ ions as additional photosensitizers, enhancing the fluorescent properties of Eu^3+^ ions. The Eu/Gd co-doped nanosheets were administered to mice via intraperitoneal (IP) injection for in vivo FI. Approximately 10 min after the IP injection, a distinct fluorescent signal was observed in enterocoelia, demonstrating the efficacy of the nanosheets as FI agents (Figure 14a,b). In particular, ultrasmall nanoparticles (d < 3 nm) are renally excretable [168] and can thus be administered via intravenous injection. Furthermore, ultrasmall Ln^3+^ ion-based nanoparticles conjugated with targeting ligands can be used for the sensitive and precise in vivo diagnosis of specific diseases such as cancer, which should be the challenging future research subjects.

For in vivo imaging, external UV light source intensity is not sufficient to excite nanoparticles inside the body for the imaging of internal organs or tumors because the penetration depth of UV in skin is poor. Zhang et al. employed an internal excitation source from Cerenkov radiation [164]. The ^89^Zr-conjugated and polyethylene glycol (PEG)-grafted ultrasmall Eu_2_O_3_ nanoparticle sample [^89^Zr-uEuNp@PEG (1 mg/mL)] was intravenously injected through the tail vein 2 weeks after CT26 tumor implantation. As shown in Figure 14c, the conventional Cerenkov radiation owing to ^89^Zr and both Cerenkov and europium radiation from ^89^Zr-uEuNp@PEG are displayed; the nanoparticles show two red emission peaks (620 and 700 nm). The red europium radiation from nanoparticles was observed with a 620 nm filter from the tumor site in mice imaging (Figure 14d).

### 4.3. Biocompatibility

**Figure 15:**
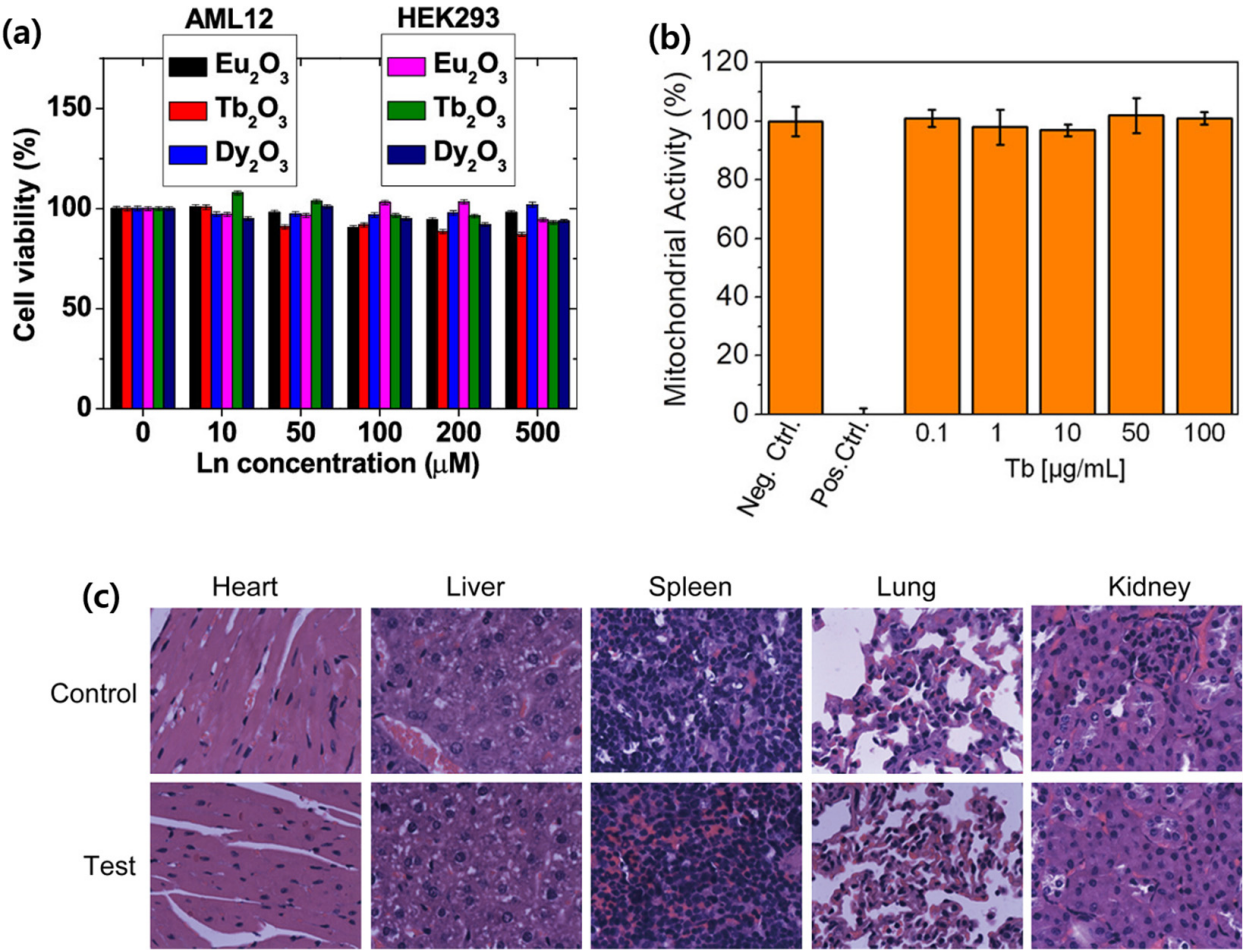
(**a**) In vitro cell viability of PAA/PDA–Ln_2_O_3_ (Ln = Eu, Tb, or Dy) nanoparticles in AML12 and HEK293 cells after 48 h of incubation, exhibiting nearly no cellular toxicity [18]. (**b**) Cell viability of PEG–TbNRs in N13 cells after 24 h of incubation, exhibiting no cellular toxicity [134]. (**c**) Hematoxylin and eosin-stained tissue sections harvested from mice before and 7 days after injection of Ir-Eu-MSN. No noticeable abnormality was observed in various organs such as the liver, spleen, heart, lung, and kidneys [9].

The good biocompatibility of Ln^3+^ ion-based nanoparticles is necessary to ensure safe diagnostic and therapeutic applications [164,166,169,170,171,172,173,174]. Compared with quantum dots, which contain toxic elements such as cadmium [175], Ln^3^⁺-based nanoparticles are less toxic [168,176]. The nanoparticles can be grafted with hydrophilic biocompatible ligands, such as PAA and PEG, to enhance their biocompatibility [18,134]. In vitro studies showed that surface-grafted Ln^3+^ ion-based nanoparticles do not significantly affect the cell viability [18,134]. As shown in Figure 15a, PAA-grafted Ln_2_O_3_ nanoparticles (Ln = Eu, Tb, and Dy) exhibited very low cytotoxicities in both alpha mouse liver12 (AML12) and human embryonic kidney293 (HEK293) cell lines, analyzed with a 3-(4,5-dimethylthiazol-2-yl)-2,5-diphenyltetrazolium bromide (MTT) assay [18]. As shown in Figure 15b, PEG–Tb nanorods (NRs) exhibited nontoxicity in N13 cells up to a concentration of 100 μg/mL (MTT assay) [134]. Therefore, surface-grafted lanthanide nanoprobes exhibited excellent cellular biocompatibility, which is suitable for in vivo applications.

As for the in vivo toxicity assay, Wu et al. conducted a histological assay of various mice organs, including the heart, lung, liver, spleen, and kidney 7 days post-injection of Ir-Eu-MSN (MSN = mesoporous silica nanoparticles, Ir–Eu = [Ir(dfppy)_2_(pic–OH)]_3_Eu·2H_2_O, dfppy = 2-(2,4-difluorophenyl)pyridine, pic–OH = 3-hydroxy-2-carboxypyridine) nanoparticles [9]. As shown in Figure 15c, the results showed no significant changes between the control and tested mice organs, indicating nontoxicity of the Ir-Eu-MSN nanoparticles. Dhanya et al. performed a histological examination of the liver and kidneys to assess the biocompatibility of PEGylated polymer capsules containing LaVO_4_:Tb^3+^ nanoparticles, one-month post-injection [170]. They found no changes in the liver and kidneys in the histological assay due to injected nanoparticles, indicating good biocompatibility in vivo.

It is crucial to consider immune responses of nanoparticles after injection. Studies showed that large nanoparticles after injection can be sequestered from circulation by phagocytic immune cells, such as monocytes and macrophages [171,173,174]. To reduce phagocytosis by immune cells, nanoparticles can be grafted with hydrophilic PEG [173,174] or can be made ultrasmall (d < 3 nm, hydrodynamic diameter < 10–15 nm) for renal excretion [177]. It was shown that PEG grafting rendered “stealth” properties against immune systems [173]. Large nanoparticles are mainly cleared out through the hepatobiliary excretion pathway with some decomposition during a long excretion time, leading to biotoxicity [177]. Therefore, Ln^3+^ ion-based nanoparticles should be made ultrasmall in diameter and should be grafted with PEG to minimize phagocytosis by immune cells and maximize their circulation and renal excretion without decomposition.

### 4.4. Targeted Imaging

**Figure 16:**
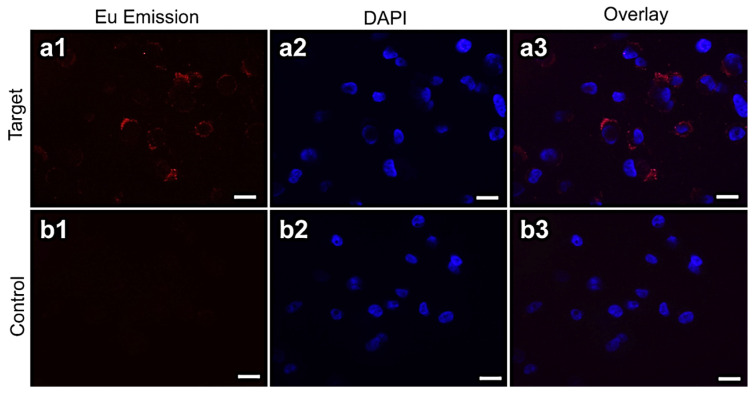
CSM images of PC-3 cells incubated with DOTA-GdVO4:4%Eu-DGEA NSs (labeled as Target, **a1**–**a3**) and DOTA-GdVO4:4%Eu (labeled as Control, **b1**–**b3**) with the concentration of 200 mg/mL for 4 h (scale bar = 20 μm). Eu red emission (λ_ex_ = 488 nm). DAPI blue emission (λ_ex_ = 405 nm) [180]. Scale bars: 20 μm.

Targeted imaging using Ln^3+^ ion-based nanoparticles represents a significant advancement in the field of diagnostic FI [178,179,180]. When functionalized with specific targeting ligands such as antibodies, aptamers, peptides, or small molecules, these nanoparticles selectively accumulate at the site of interest such as cancer, enhancing the specificity and sensitivity of imaging modalities and early detection of diseases [181]. In addition, by combining FI with magnetic resonance imaging (MRI) or computed tomography (CT), researchers can achieve a more comprehensive understanding of disease status while overcoming the limitations of each modality, leading to a more accurate and detailed diagnosis of disease [182]. For example, Hu et al. developed ^64^Cu-DOTA-GdVO4:4%Eu-DGEA (DOTA = dodecane tetraacetic acid, DGEA = Asp-Gly-Glu-Ala peptide for integrin α_2_β_1_ targeting) nanosheets as multimodal imaging agents that simultaneously possessed radioactivity, fluorescence, and paramagnetic properties [180]. An intense Eu^3+^ red signal was observed on the surface and in the cytoplasm of human prostate cancer (PC-3) cells upon 488 nm laser excitation using confocal scanning microscopy (CSM), confirming the integrin α_2_β_1_ specificity of DGEA-conjugated nanosheets (Figure 16a1). The nuclei of the PC-3 cells were stained with the blue fluorescent dye 40, 6-diamidino-2-phenylindole (DAPI), as shown in Figure 16a2. The overlay of Eu^3+^ and DAPI emission (Figure 16a3) clearly shows the distribution of DGEA-conjugated nanosheets, i.e., on the cell surfaces and inside the cells. The control experiment that was conducted by incubating DOTA-GdVO4:4%Eu (no DGEA ligands) with PC-3 cells under identical conditions showed nearly no red signals on the surface of the PC-3 cells due to the absence of specific integrin α_2_β_1_ targeting ligands (Figure 16b1–b3).

### 4.5. Theranostic Applications

**Figure 17:**
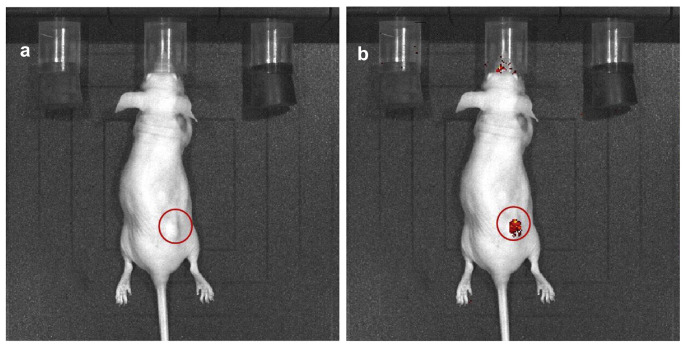

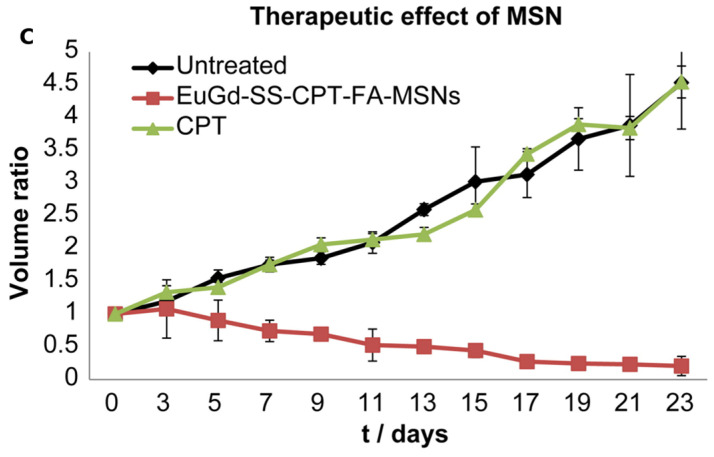
In vivo PL imaging of mice after subcutaneous injection (**a**) without and (**b**) with EuGd-MSNs (Eu^3+^:Gd^3+^ = 1:1) at an excitation wavelength of 430 nm. Red circles indicate tumor. (**c**) Tumor growth curves for HeLa tumor cell-bearing mice from 0 to 23 days; error bars were determined using 3 mice in each group [187].

Theranosis is an innovative approach that integrates diagnosis and therapy into a unified platform [183]. Nanoparticles are an ideal system for theranosis because specific targeting ligands and drugs can be easily conjugated to them. The Ln^3+^ ion-based nanoparticles can provide theranosis through their FI combined with targeted drug delivery [184,185,186,187]. For example, Chan et al. developed multifunctional europium (Eu)- and gadolinium (Gd)-conjugated mesoporous silica nanoparticles (MSNs) that serve as a theranostic platform for cancer treatment [187]. The nanoparticles, owing to both fluorescence and magnetic properties, enabled dual-mode FI and MRI. Folic acid (FA) was grafted onto the surface of the EuGd-MSNs to allow for the specific binding to folate receptors that are overexpressed on cancer cells. Additionally, camptothecin (CPT), as an anticancer drug, was attached to the MSNs. In vivo FI studies demonstrated that the EuGd-SS-CPT-FA-MSNs effectively targeted tumors; the control group without nanoparticles showed no red fluorescence from Eu (Figure 17a), whereas the treatment group with nanoparticles showed red fluorescence from Eu (Figure 17b). Figure 17c illustrates that tumors in the treatment group nearly disappeared after 17 days, while tumors in the control groups continued to grow. The ineffectiveness of the anticancer drug CPT alone in inhibiting tumor growth confirms the potential of these multifunctional nanoparticles as a theranostic platform that combines FI and targeted therapy of cancer.

### 4.6. Comparison to MRI and CT

Compared to MRI and CT, FI offers unique advantages for imaging applications. FI provides high sensitivity and high resolution [188,189,190], allowing for the detection of fluorescent probes at extremely low concentrations (pico- to femto-molar) [191]. FI excels over MRI and CT given its higher resolution, higher sensitivity, and lower cost [188,189,190,192]. These properties make FI valuable for tracking drugs and nanoparticles within biological systems. However, FI has limitations in tissue penetration depth owing to light absorption and scattering by skin [193]. This limitation restricts its applicability for deep tissue imaging compared to MRI and CT. In contrast, MRI and CT offer superior anatomical details and are well suited for deep tissue imaging, with MRI providing a noninvasive approach [194]. Safety is a critical consideration in imaging techniques. FI is generally considered safe, as it does not utilize ionizing radiation. MRI and CT contrast agents are typically safe but may occasionally lead to allergic reactions or complications in patients with kidney problems [195]. On the other hand, CT involves exposure to ionizing radiation, which raises concerns, particularly regarding repeated exposure [196]. Therefore, the choice of these imaging modalities depends on specific applications.

## 5. Effects of Physical Conditions on PL Intensity

For biomedical applications, Ln^3^⁺ ion-based nanoparticles should be grafted with hydrophilic biocompatible ligands such as PAA or PEG to be stable as colloids in aqueous media under physiological conditions (1 atm, 20~40 °C, pH = 6~8, 1~20 mM glucose, 0.9% sodium chloride solution). Note that the PL intensity depends on pH [197,198] and temperature [199,200,201]. It decreases with increasing pH value because of the increasing number of –OH groups on nanoparticle surfaces, which participate in fluorescence quenching. It decreases with increasing temperature because of thermal deactivation (or thermal quenching) of emitting states of Ln^3+^ ions and the triplet excited state of photosensitizing organic ligands. Under physiological conditions, the–OH group quenching can be minimized by protecting nanoparticle surfaces through surface-grafting with hydrophilic biocompatible ligands or may be avoided through the core–shell structure, whereas the thermal quenching may be inevitable.

## 6. Conclusions and Outlook

Despite their outstanding fluorescent properties in the visible region, such as nearly constant transition energies, long emission lifetimes, atomic-like sharp transitions, large Stokes shifts, high photostability, and resistance to photobleaching, the applications of Ln^3+^ ion-based nanoparticles have been hindered by their low QYs, owing to the selection rule-forbidden 4f–4f electronic transitions of Ln^3+^ ions. However, advances in the development of Ln^3+^ ion-based nanoparticles with high QYs were successfully achieved through photosensitization using organic ligands or host matrices, which absorb incoming photons and transfer energy to Ln^3+^ ions. This led to a pivotal breakthrough in FI applications of the Ln^3+^ ion-based nanoparticles. The high QYs achieved through photosensitization, combined with their excellent fluorescent properties, make these nanoparticles promising next-generation fluorescent probes, superior to conventional dyes and QDs.

Among photosensitized Ln^3+^ ion-based nanoparticles, those containing Ln^3+^ ions (Ln = Eu, Tb, and Dy) are especially useful, owing to their unique intense red, green, and yellow emissions, respectively. In particular, Dy^3+^ ion-based nanoparticles exhibit promising potential as white phosphors in single-phase systems by adjusting the ratio between the yellow and blue light emission intensities.

This review focused on various strategies for improving the QY of Ln^3+^ ion-based nanoparticles based on photosensitization, i.e., the doping of Ln^3+^ ions into host matrices or capping with organic ligands, which served as photosensitizers. In addition, we discussed several strategies for further improving the QY of photosensitized Ln^3+^ ion-based nanoparticles: (i) core–shell structure, i.e., the surface passivation of the core nanoparticle with an optically transparent thin-layer shell; (ii) the co-doping of Eu^3+^/Tb^3+^ ions into the host matrix to achieve a higher QY of Eu^3+^ ions through the energy transfer from Tb^3+^ ions. In addition, color tuning between green and red was achieved by varying the Eu^3+^/Tb^3+^ ratio; (iii) the co-doping of secondary metal ions such as Gd^3+^ and Ce^3+^ ions, which significantly enhances the QY of Ln^3+^ ions by facilitating the energy transfer from the host matrix and acting as photosensitizers (synergistic effect); (iv) the co-doping with Li^+^, Na^+^, K^+^, and Ba^2+^ ions, which breaks the parity selection rule, changes the local crystal field, reduces the lattice strains, and modifies the crystal phase, leading to further enhanced QYs. A high QY is essential for State-of-the-Art applications of Ln^3+^ ion-based nanoparticles as fluorescent probes.

In addition to the high QYs achieved through photosensitization, the Ln^3+^ ion-based nanoparticles can be used for background autofluorescence-free signal detection through time-resolved FI, enabling the detection of only Ln^3+^ ion signals in biological samples. This time-resolved FI, which is not viable for organic dyes and QDs, is possible because of the long fluorescence lifetimes of Ln^3+^ ions (milli- to microseconds), whereas the background autofluorescence from biological samples typically occurs on the nanosecond timescale. Using time-resolved FI, ultrasensitive detection at picomolar level concentrations may be possible, enabling the early diagnosis of diseases.

As discussed in this review, photosensitized Ln^3+^ ion-based nanoparticles demonstrated remarkable potential for FI applications as in vitro and in vivo fluorescent probes. To advance and extend their applications, their QYs should be further improved. This goal requires the development of new photosensitizers, the optimization of co-doping elements, and surface passivation. This action will maximize the potential of the Ln^3+^ ion-based nanoparticles as ultrasensitive fluorescent probes, paving the way for next-generation State-of-the-Art applications. In particular, the development of ultrasmall Ln^3+^ ion-based nanoparticles will be extremely useful for in vivo applications because of their renal excretion ability, which enables their intravenous injection for specific disease detection (diagnosis) and drug delivery (therapy) after conjugating them with targeting ligands. In addition, their short residence time in the body, owing to their renal excretion, minimizes their biotoxicity. The excellent performance of the photosensitized Ln^3+^ ion-based nanoparticles reviewed in this paper highlights their promising potential as fluorescent probes in future applications. This review provides valuable insight into the design and application of Ln^3+^ ion-based nanoparticles with high QYs achieved via photosensitization with potential use in biomedical FI.

## Figures and Tables

**Table 1:** Photosensitized Ln^3+^ ion-based nanoparticles with high QYs.

Nanoparticle	Size	Photosensitizer (Host Matrix or Co-Doping Ion or Organic Ligand)	λ_ex_ (nm)	QY (%)	Ref.
LaF_3_: Ce, Tb	12 nm	Ce^3+^	252	Tb = 60	[24]
CaMoO_4_: Tb; CaMoO_4_: Eu	50–70 nm	(MoO_4_)^2−^	Tb = 300 Eu = 320	Tb = 52, Eu = 82	[25]
YOSe: Tb; YOSe: Eu	22.35 ± 1.79 nm, 27 ± 0.11 nm	YOSe (O^2−^ and Se^2−^)	Tb = 350 Eu = 350	Tb = 57, Eu = 53	[26]
LaMoBO_6_: Tb^3+^, Eu^3+^, Bi^3+^		Bi^3+^, Tb^3+^	487	Tb + Eu = 92	[27]
CaNb_2_O_6_: Dy^3+^		NbO_6_	265	Dy = 71.2	[28]
NaYF_4_: Tb@NaYF_4_: Ce; NaYF_4_: Eu@NaYF_4_: Ce; NaYF_4_: Ce/Dy@NaYF_4_		Ce^3+^	250	Tb = 90; Eu = 75; Dy = 50	[29]
LaNbO_4_: 0.01Tb^3+^		NbO_4_^3−^	261	Tb = 43.2	[30]
LaPO_4_: Ce, Tb		Ce^3+^	266	Tb = 73, Tb + Ce = 86	[31]
LaPO_4_: Ce, Tb	9–12 nm	Ce^3+^	273	Tb = 70, Tb + Ce = 90	[32]
LaPO_4_: Ce^3+,^ Tb^3+^	2.7 nm	Ce^3+^	312	Tb = 74	[33]
LaPO_4_: Ce, Tb	5–6 nm	Ce^3+^	266	Tb = 42, Tb + Ce = 61	[34]
CePO_4_: Tb/LaPO_4_	8 nm	Ce^3+^	277	Tb = 70, Tb + Ce = 80	[21]
LaF_3_: Ce, Tb/LaF_3_	9 nm	Ce^3+^	282	Tb + Ce = 54	[35]
CePO_4_: Tb/LaPO_4_		Ce^3+^	277	Tb = 70, Tb + Ce = 90	[20]
NaYF_4_: Ce^3+^, Tb^3+^	2.7 nm	Ce^3+^	290	Tb = 54.8	[36]
Sr_3_Y_0.99_Dy_0.01_(PO_4_)_3_	42.6 nm	PO_4_^3−^	350	Dy = 96	[37]
K_3_Y(PO_4_)_2_: Dy^3+^	200–500 nm	PO_4_^3−^	350	Dy = 13	[38]
YVO_4_: 0.05%Dy	4 × 2 × 1 mm	VO_4_^3−^	340	Dy = 14.1	[39]
(Gd_0.98_Dy_0.02_)_2_O_3_	438 nm	Gd^3+^	275	Dy = 61.2	[40]
KSrVO_4_: Dy^3+^	36.33 nm	VO_4_^3−^	390	Dy = 39	[41]
NaGdF_4_: 0.2% Dy^3+^	100–400	Gd^3+^	273	Dy = 88.86	[42]
NaGd(WO_4_)_2_: Dy^3+^	20 × 40 mm^2^	Gd^3+^ and WO_4_^2−^	388	Dy = 63	[43]
Ba_1-x-y_Dy_x_K_y_WO_4_	6–14 µm	WO_4_^2−^	351	Dy = 67	[44]
YVO_4_: 2Dy: 2Ba	29 nm	VO_4_^3−^	320	Dy = 47	[45]
Gd_2_O_3_: Dy^3+^	47 nm	Gd^3+^	274	Dy = 21.6	[46]
YVO_4_: Eu^3+^; YVO_4_: Dy^3+^	1 μm	VO_4_^3−^	Eu = 291 Dy = 283	Eu = 70; Dy = 62	[47]
Gd_2_O_3_: Eu^3+^	20 nm × 60 µm	Gd^3+^	252	Eu = 90	[48]
YVO_4_: Eu	20 nm	VO_4_^3−^	272	Eu = 54	[49]
GdOF: Eu^3+^; GdOF: Tb^3+^		Gd^3+^	Eu = 255 Tb = 235	Eu = 89.3; Tb = 71.2	[50]
Sr_0.85_Ce_0.05_Tb_0.1_F_2.15_	5–6 nm	Ce^3+^	310	Tb = 79.7	[51]
LaPO_4_: Ce, Tb	2.9 ± 0.2 nm	Ce^3+^	272	Tb = 50	[52]
Y_2_O_3_: Eu^3+^; Y_2_O_3_: Tb^3+^	(80–120 nm) × (2–5 μm)	O^2−^	Eu = 254 Tb = 305	E = 86; Tb = 71	[53]
KGdF_4_: 5% Ce^3+^, 4% Tb^3+^	215 nm	Ce^3+^ and Gd^3+^	260	Ce + Tb = 64	[54]
GdVO_4_: Eu^3+^/Tb (BA)_3_Phen; GdVO_4_: Dy^3+^/Eu (BA)_3_Phen	400–500 nm	(BA)_3_Phen and VO_4_^3−^	290	Eu + Tb = 62.9; Dy + Eu = 49.2	[55]
Eu-Gd oxide	2.1 nm	Gd^3+^		Eu = 33	[56]
Y_2_O_3_: Eu^3+^_0.1_; Y_2_O_3_: Eu^3+^_0.1_, Tb^3+^_0.0025_; Y_2_O_3_: Eu^3+^_0.1_, Tb^3+^_0.0025_, Ce^3+^_0.001_	2.873 µm	O^2−^; Tb^3+^; Ce^3+^	254	Eu = 75.4; Eu = 88.2; Eu = 85.1	[57]
GdNbO_4_: 0.001Eu^3+^; GdNbO_4_: 0.004Tb^3+^	30–50 nm	NbO_4_^3−^ and Gd^3+^	Eu = 262 Tb = 257	Eu = 59.7; Tb = 59.1	[58]
NaTbF_4_: 2%Eu		Tb^3+^	377	Eu = 69.9	[59]
LaPO_4_: Eu	200–360 nm	Polyvinylpyrrolidone (PVP)	270	Eu = 94.6	[17]
CaMoO_4_: Eu, 10%Gd		Gd^3+^ and MoO_4_^2−^	277	Eu = 80	[60]
Gd_2_O_3_: Eu^3+^	42.17 ± 0.27 nm	Gd^3+^ and O^2−^	254	Eu = 64.83 ± 0.018	[61]
BHHCT-Eu^3+^-DPBT	<50 nm	BHHCT	406	Eu = 66	[12]
Gd_2_O_3_: Eu@silica	150 nm	Gd^3+^ and O^2−^	255	Eu = 56	[62]
CaMoO_4_: Eu^3+^	3.4 nm	MoO_4_^2−^	290	Eu = 85.3	[63]
Ca_2_SiO_4_: Eu^3+^	20 nm	O^2−^	270, 397	Eu = 87.95	[64]
Gd_4.67_ Si_3_O_13_/Eu^3+^		Gd^3+^ and O^2−^	275, 394, 406	Eu = 89.2 (394 nm) Eu = 82.1 (406 nm)	[65]
LaOF: 5% Eu^3+^	15.3 ± 1.7 nm	O^2−^	270	Eu = 100	[66]
Gd_2_O_3_: Eu^3+^	84.94 ± 2.08 nm	Gd^3+^ and O^2−^	254	Eu = 53.32 ± 0.02	[67]
Y_2_O_3_: Eu^3+^	20 nm	O^2−^	245, 395	Eu = 60	[68]
Eu_2_O_3_; Tb_2_O_3_	2.3 ± 0.1 nm; 2.2 ± 0.1 nm	2,6-pyridine dicarboxylic acid (PDA)	Eu=287 Tb = 286	Eu = 87.6; Tb = 73.6	[18]
LaF_3_: Eu^3+^	11.5	Thenoyltrifluoroacetone	350	Eu = 61.6	[69]
YVO_4_: Eu^3+^	3.6 nm	VO_4_^3−^	310	Eu = 62	[70]
GdPO_4_: Tb^3+^, Ce^3+^	(15–20 nm) × (600–700 nm)	Gd^3+^, Ce^3+^	280	28	[71]
CaMoO_4_: Eu^3+^	4 nm	MoO_4_^2−^	280 nm	Eu = 75	[72]
Y_1.9_O_3_: Eu_0.1_^3+^	6 nm	O^2−^	246	Eu = 90	[73]
Tb_x_ZnAl_2−x_O_4_ (X = 3.56%)	2.2 ± 0.5 nm	1,1,1,5,5,5-Hexafluoro-2,4-pentanedione (hfacac)	320	Tb = 37	[74]
NaYF_4_: Ce/Tb	20 to 40 nm	Ce^3+^	290	Tb + Ce = 56.4	[75]
Ca_4_LaO (BO_3_)_3_: Tb^3+^	200–500 nm	O^2−^	271	Tb = 98	[76]
YOF: 0.01Tb^3+^; YOF: 0.01Tb^3+^, 0.005Eu^3+^	45–85 nm	O^2−^ and Tb^3+^	241	Tb = 100; Tb + Eu = 100	[77]
Ba_3_Y_4_O_9_: Tb^3+^	43 nm	O^2−^	292	Tb = 93.04	[78]
La_0.9_Tb_0.1_F_3_	20–25 nm	3,3′-((butane 1,4-diylbis(azanediyl)) bis(carbonyl))bis(2-hydroxybenzoic acid)	337	Tb = 29	[79]
ZnAl_2_O_4_: Tb^3+^	2.2 ± 0.5 nm	hexafluoroacetylacetonate	320	Tb = 39	[80]
CePO_4_: Tb	7.6 nm × 78 nm	Ce^3+^	274	Tb = 36	[81]
CePO_4_: Tb	(4–8 nm) × (100–200 nm)	Ce^3+^	286	Tb + Ce = 40	[82]
Gd_2_O_3_: Tb^3+^	3.5 nm	Gd^3+^	275	Tb = 52 ± 5	[83]
Tb_0.05_Y_0.95_PO_4_; Eu_0.05_Y_0.95_PO_4_	110 ± 20 nm; 123 ± 15 nm	O^2−^	Eu = 395 Tb = 351	Tb = 80.7; Eu = 62.9	[84]
L-LTbH: Gd		2,2′-(((2,4,6-Trimethyl-1,3-phenylene) bis(methylene)) bis(oxy)) dibenzoic acid	365	Tb = 33	[85]
GdF_3_: Eu^3+^	30–35 nm	[2,3-f] Pyrazino [1,10] phenanthroline-2,3-dicarboxylic acid (PPDB^2−^)	360	Eu = 34	[86]
LaPO_4_: Eu^3+^/LaPO_4_	6.5 ± 1.3 nm	O^2−^	250	Eu = 50	[87]
Lu_2_O_3_: Eu	30 nm	O^2−^	255	Eu = 90	[88]
(Gd_0.952_Dy_0.008_Eu_0.04_)_2_O_3_		O^2−^, Gd^3+^, Dy^3+^	275	Eu + Dy = 72.1, mostly from Eu	[89]
Y_2_Zr_2_O_7_: Eu^3+^	77.2 nm	O^2−^	264	E = 88.2	[90]
